# LMP7-Specific Inhibitor M3258 Modulates the Tumor Microenvironment of Triple-Negative Breast Cancer and Inflammatory Breast Cancer

**DOI:** 10.3390/cancers17111887

**Published:** 2025-06-04

**Authors:** Xuemei Xie, Jangsoon Lee, Ganiraju C. Manyam, Troy Pearson, Gina Walter-Bausch, Manja Friese-Hamim, Sheng Zhao, Julia Jabs, Angela A. Manginelli, Nadine Piske, Thomas Mrowiec, Corinna M. Wolf, Bharat S. Kuntal, Debu Tripathy, Jing Wang, Michael P. Sanderson, Naoto T. Ueno

**Affiliations:** 1 Section of Translational Breast Cancer Research, The University of Texas MD Anderson Cancer Center, Houston, TX 77030, USA; xxie@cc.hawaii.edu (X.X.); jlee@cc.hawaii.edu (J.L.); tpearson@mdanderson.org (T.P.); debu.tripathy@sbcglobal.net (D.T.); 2 Department of Breast Medical Oncology, The University of Texas MD Anderson Cancer Center, Houston, TX 77030, USA; 3 Cancer Biology Program, University of Hawaiʻi Cancer Center, Honolulu, HI 96813, USA; bsingh@cc.hawaii.edu; 4 Department of Bioinformatics and Computational Biology, The University of Texas MD Anderson Cancer Center, Houston, TX 77030, USA; gcmanyam@mdanderson.org (G.C.M.); jingwang@mdanderson.org (J.W.); 5 Institute Associate Scientist IV Belfer Neurodegeneration Consort, The University of Texas MD Anderson Cancer Center, Houston, TX 77030, USA; 6 Merck Healthcare KGaA, 64293 Darmstadt, Germany; gina.walter-bausch@merckgroup.com (G.W.-B.); sheng.zhao@sanofi.com (S.Z.); julia.jabs@merckgroup.com (J.J.); angela.manginelli@merckgroup.com (A.A.M.); nadine.piske@merckgroup.com (N.P.); thomas.mrowiec@merckgroup.com (T.M.); corinna.wolf@merckgroup.com (C.M.W.); michaelsand@hotmail.com (M.P.S.); 7 Merck KGaA, 64293 Darmstadt, Germany; manja.friese-hamim@merckgroup.com; 8 Sanofi Deutschland, 60311 Frankfurt am Main, Germany; 9 Molecular Partners AG, 8952 Zurich-Schlieren, Switzerland

**Keywords:** LMP7, M3258, TNBC, IBC, tumor microenvironment

## Abstract

Triple-negative breast cancer (TNBC) and inflammatory breast cancer (IBC) are the most aggressive molecular subtypes of breast cancer. Poor clinical outcomes highlight the pressing need to discover novel targets for the effective treatment of these diseases. LMP7 (β5i/PSMB8), a proteolytic subunit of the immunoproteasome, is implicated in the pathogenesis of multiple myeloma, autoimmune and inflammatory diseases, and inflammation-related cancers. However, the role of LMP7 in TNBC and IBC remains poorly characterized. Here, we evaluated the function of LMP7 in TNBC and IBC using the selective LMP7 inhibitor M3258. In human TNBC patient samples, LMP7 expression correlated strongly with CD8^+^ T cell infiltration and activation markers. M3258 inhibited LMP7 activity, reduced viability, and induced apoptosis in TNBC/IBC cell lines in vitro. In a novel immunocompetent in vivo model of TNBC/IBC, M3258 reduced tumor growth and the tumor abundance of M2 macrophages. Additionally, M3258 activated tumor-infiltrating CD8^+^ T cells and suppressed the expression of specific inflammatory pathway gene signatures in immune cells. Co-culture with M2 macrophages enhanced the invasiveness of TNBC/IBC cells, which was effectively suppressed by M3258 treatment. Our results demonstrate for the first time that LMP7 shapes the pro-tumorigenic microenvironment of TNBC/IBC, in part by modulating the pathogenic role of M2 macrophages. These findings suggest that LMP7 may represent a novel target for therapeutic intervention in TNBC/IBC.

## Simple Summary

Triple-negative breast cancer (TNBC) and inflammatory breast cancer (IBC) are highly aggressive breast cancer subtypes with poor clinical outcomes, which emphasizes the need for new therapeutic targets for these diseases. LMP7, a subunit of the immunoproteasome, has been linked to inflammatory diseases and inflammation-driven cancers, but its role in TNBC and IBC remains unknown. This study assessed the function of LMP7 in TNBC and IBC models using the selective LMP7 inhibitor M3258. M3258 suppressed the viability of TNBC/IBC cells in vitro by inhibiting LMP7 activity and significantly inhibited tumor growth in a humanized immunocompetent mouse model. M3258 also reduced M2 macrophage abundance in the TNBC/IBC tumors and induced activation of tumor-infiltrating CD8^+^ T cells. Additionally, M3258 suppressed M2 macrophage-promoted invasive behavior of TNBC/IBC cells. These findings demonstrate that LMP7 plays a key role in shaping the tumor microenvironment and highlight its potential as a novel therapeutic target for TNBC/IBC.

## 1. Introduction

The immunoproteasome (IP) has emerged as a promising therapeutic target in hematological cancers and inflammatory diseases. The IP and constitutive proteasome are large proteolytic complexes that degrade ubiquitinated proteins to create peptides for antigen presentation [1,2]. The IP is predominantly expressed in hematolymphoid cells and cancers such as multiple myeloma (MM) [3] but can be induced in cells of non-hematolymphoid origin by inflammatory conditions, such as exposure to IFNγ or TNFα [4]. The presence of alternate proteolytic subunits in the IP, as compared to the constitutive proteasome, results in the generation of a distinct repertoire of degraded peptides, which are efficiently complexed on major histocompatibility complex class I for presentation to CD8^+^ T cells during adaptive immunity [5].

Pan-proteasome inhibitors (pan-PIs), such as bortezomib, target the catalytic activity of several proteolytic subunits of the IP and the constitutive proteasome [6]. Pan-PIs have demonstrated robust clinical efficacy in MM, which led to their approval for the treatment of MM [7]. In contrast, despite a wealth of clinical testing, pan-PIs have demonstrated only limited activity in solid tumors [8]. This may reflect a lower dependency of solid tumor cells on proteasome function compared to MM cells [9]. Additionally, the diverse toxic effects associated with pan-PIs, which may stem from inhibition of the broadly expressed constitutive proteasome in healthy tissues, may limit the dose of pan-PIs to levels below that required for therapeutic activity in solid tumors [10].

Considerable attention has turned to the potential of selective IP inhibitors to avoid the toxic effects associated with pan-PIs [11]. In particular, the IP subunit large multifunctional peptidase 7 (LMP7/β5i/PSMB8) has been implicated in the pathogenesis of MM and autoimmune diseases [12]. M3258, an orally bioavailable, highly selective, potent, and reversible inhibitor of LMP7, has exhibited a favorable non-clinical safety profile and antitumor activity in various in vivo MM models [13,14,15]. In addition, dual LMP2/LMP7 inhibitors such as KZR-616 and ONX-0914 have demonstrated promising activity in experimental models of autoimmune diseases [7,9,16,17]. However, while preclinical studies have assessed the effects of LMP7 inhibition in models of colorectal cancer [18] and prostate cancer [19], the value of LMP7 inhibitors in other solid tumors with high unmet therapeutic need has not been extensively characterized.

Triple-negative breast cancer (TNBC) is characterized by the absence of the estrogen receptor and progesterone receptor, as well as the absence or low expression of human epidermal growth factor receptor 2. TNBC accounts for approximately 15% to 20% of all breast cancer cases. TNBC has a more aggressive phenotype and worse prognosis than other breast cancer subtypes [20], with a mortality rate of 40% within the first 5 years following diagnosis [21,22,23,24]. Inflammatory breast cancer (IBC) is characterized by the rapid onset of inflamed skin, *peau d’orange*, edema, or warm breasts, with or without an underlying palpable mass [25]. IBC can occur across the different classical breast cancer subtypes and represents 1% to 6% of breast cancer cases in the United States [26] and 10% to 15% in North African countries [27,28]. The overall 5-year survival rate for patients with IBC is less than 30% [29]. Inflammation is a common feature of TNBC and is central to the pathogenesis of IBC. Overexpression of LMP7 has been described in a subset of TNBC patients and has been associated with elevated CD8^+^ T cell infiltration and activation [30,31]. While pan-PIs have yielded limited clinical efficacy in unselected breast cancer patients [32,33], selective LMP7 inhibitors may hold promise in TNBC exhibiting elevated LMP7 expression. However, to date, the precise role of LMP7 in TNBC and IBC remains poorly characterized.

In this study, we used the selective LMP7 inhibitor M3258 to investigate the role of LMP7 in the pathogenesis of TNBC/IBC using preclinical models. M3258 exhibited direct antitumor activity against TNBC/IBC cells in vitro. M3258 also reshaped the tumor microenvironment (TME) of an in vivo model of TNBC/IBC by reducing the tumor abundance of M2 macrophages and inducing the activation of CD8^+^ T cells. Meanwhile, M3258 suppressed M2 macrophage-induced invasion of TNBC/IBC cells. Our results demonstrate for the first time that LMP7 plays an important role in promoting an inflammatory TME and enhancing the proliferation and invasiveness of TNBC/IBC cells, in particular by modulating the pathogenic role of M2 macrophages. Our findings reveal insights into the role of LMP7 in TNBC/IBC and suggest that inhibition of LMP7 may represent an attractive therapeutic strategy in these diseases.

## 2. Materials and Methods

### 2.1. Immunohistochemistry (IHC) Staining of Human TNBC Tumors

Formalin-fixed paraffin-embedded tissue blocks obtained from 30 surgically resected human TNBC samples were subjected to IHC analysis to evaluate the expression of LMP7, CD8, and PD-L1. The analysis was performed by the external commercial provider Indivumed GmbH (Hamburg, Germany). All tumor samples were procured from Indivumed GmbH under Institutional Review Board approval and informed consent in Russia or Ukraine, respectively. The only inclusion criteria for the study samples were primary TNBC and stage I, II, or III TNBC.

Staining of serial sections was performed in a predefined order as follows: PD-L1, hematoxylin and eosin [H&E], LMP7, CD8, isotype control LMP7, isotype control CD8, and isotype control PD-L1. For PD-L1 and LMP7, IHC was implemented on the BenchMark Ultra staining platform (Roche Tissue Diagnostics/Ventana Medical Systems, Oro Valley, AZ, USA) using rabbit monoclonal anti-PD-L1 antibody clone SP263 (790-4905; Roche Tissue Diagnostics) and rabbit monoclonal anti-LMP7 antibody clone EPR14482(B) (0.4 µg/mL; ab180606; Abcam, Cambridge, UK). Slides were deparaffinized within the staining instrument and immunostained using the OptiView DAB IHC Detection Kit for PD-L1 staining and the ultraView Universal DAB Detection Kit (Roche Tissue Diagnostics) for LMP7 staining. For CD8, IHC was implemented on the Discovery XT staining platform (Roche Tissue Diagnostics) using rabbit monoclonal anti-CD8 antibody clone SP16 (0.1667 µg/mL; NU740-5UC; DCS-Innovative Diagnostik-Systeme, Hamburg, Germany). For IHC, slides were deparaffinized within the staining instrument and immunostained using the Discovery ChromoMap DAB Kit (Roche Tissue Diagnostics). After staining, the slides were manually washed using hot tap water supplemented with detergent, followed by tap water, and then ddH_2_O in a final step. For dehydration, the slides were transferred to an ascending ethanol series. The slides were then transferred to xylene for 2 min and automatically cover-slipped in Pertex.

### 2.2. Digital Image Analysis of Immunostained TNBC Tumor Samples

Immunostaining was quantified by digital image analysis using HALO version 3.3 (Indica Labs, Albuquerque, NM, USA). Scans of serial sections stained for PD-L1, H&E, LMP7, and CD8 were registered. Areas of tumor cells and tumor stroma were separated on the H&E scan using a customized random forest tissue classifier. Subsequently, the tumor cell area and tumor stroma area were transferred from the H&E scan to all 3 IHC scans of the respective case. Co-registration quality was assessed for each sample, and 29 of 30 samples passed visual quality control. On each of the IHC scans, cells were segmented using customized image analysis settings for the respective marker based on the HALO Multiplex IHC version 2.3.4 algorithm. LMP7 and CD8 positivity were assessed in the nucleus and cytoplasm, whereas PD-L1 positivity was assessed exclusively in the membrane. Cells were classified as either negative or positive for CD8 and as negative, weakly, moderately, or strongly positive for LMP7 and PD-L1. Statistical analyses were performed as described in Section 2.12 below.

### 2.3. Cell Lines and Reagents

The breast cancer cell lines used in this study included SUM-149 PT (TNBC/IBC; Asterand Bioscience, Detroit, MI, USA), FC-IBC02 (TNBC/IBC; Fox Chase Cancer Center, Philadelphia, PA, USA), BCX-010 (TNBC/IBC; MD Anderson Cancer Center, Houston, TX, USA), and HCC1187 (TNBC; American Type Culture Collection [ATCC], Manassas, VA, USA). HCC1187 cells were maintained in RPMI 1640 medium (Life Technologies Inc., Grand Island, NY, USA) supplemented with 10% fetal bovine serum (FBS, MilliporeSigma, Billerica, MA, USA) and 1% antibiotic/antimycotic (GenDEPOT, Katy, TX, USA). SUM-149 PT, FC-IBC02, and BCX-010 cells were maintained in Ham’s F-12 medium (Life Technologies Inc.) supplemented with 5% FBS, 1% antibiotic/antimycotic, 5 µg/mL insulin (Thermo Fisher Scientific Inc., Waltham, MA, USA), and 1 µg/mL hydrocortisone (Sigma-Aldrich, St. Louis, MO, USA). The human pancreatic cancer cell line SW 1990 (ATCC) and human lung cancer cell line A549 (ATCC) were both maintained in DMEM (Thermo Fisher Scientific Inc.) supplemented with 10% FBS, 2 mM GlutaMAX Supplement (Thermo Fisher Scientific Inc.), and 1 mM sodium pyruvate (MilliporeSigma). The human monocytic cell line THP-1 (ATCC) was maintained in RPMI 1640 medium supplemented with 10% FBS, 1% antibiotic/antimycotic, and 1% pyruvate (Sigma-Aldrich). All cell lines were validated by DNA typing at the MD Anderson Cancer Center Characterized Cell Line Core (Houston, TX, USA) and confirmed to be free of *Mycoplasma* infection.

### 2.4. Assessment of LMP7 Proteolytic Activity

The effect of M3258 on LMP7 proteolytic activity in breast cancer cell lines was tested in vitro. Breast cancer cells were seeded at 10,000 to 20,000 cells/well in 96-well black plates in the presence or absence of recombinant human IFNγ (100 IU/mL, R&D Systems, Minneapolis, MN, USA) for 22 h at 37 °C. The next day, cells were treated with M3258 at the desired concentrations for 2 h at 37 °C. Fifty microliters of lysis buffer containing 30 µM LMP7 peptidic substrate (Ac-ANW)2R110 (Biomol, Hamburg, Germany) was added to each well, and the plates were incubated for 1 h at 37 °C. Absorbance was measured at 535 nm. LMP7 proteolytic activity inhibition graphs for determining IC_50_ values were generated using GraphPad Prism 10 (Boston, MA, USA).

### 2.5. In Vitro Cell Viability Assay

The effect of M3258 on the viability of TNBC/IBC cells was assessed using the CellTiter-Blue cell viability assay (Promega Corporation, Fitchburg, WI, USA). Cells were seeded at 1000 to 3000 cells/well in 96-well plates in the presence or absence of recombinant human IFNγ (100 IU/mL). The next day, M3258 (0 to 50 µM) was applied, and the cells were cultured for a further 72 h. CellTiter-Blue reagent was then added to each well, and absorbance was measured at 595 nm. Growth inhibition graphs for determining IC_50_ values were generated using GraphPad Prism 10.

### 2.6. Caspase 3/7 Activity Measurement

Caspase 3/7 activity in breast cancer cell lines was measured using the Caspase-Glo 3/7 assay (Promega Corporation). Cells were seeded at 10,000 to 20,000 cells/well in 96-well white plates in the presence or absence of recombinant human IFNγ (25 IU/mL) and cultured overnight. M3258 was then applied at the desired concentrations, and the cells were cultured for a further 24 h. The culture supernatants were then discarded, and the Caspase-Glo 3/7 substrate solution was added to each well. Following a 1-h incubation at room temperature, luminance signals were measured using a VICTOR X3 plate reader (PerkinElmer, Waltham, MA, USA).

### 2.7. Western Blotting

The effects of IFNγ and M3258 on the expression of proteasome subunits in breast cancer cell lines were examined by Western blotting. One million cells were seeded in 10-cm plates and cultured overnight in the presence or absence of recombinant human IFNγ (100 IU/mL). The cells were then cultured in the desired concentration of M3258 for 48 h. Proteins were then extracted and processed for Western blot analysis. Proteins of interest were detected using the following primary antibodies at 1:1000 dilution: anti-LMP7 (ab3329; Abcam), anti-PSMB9 (LMP2) (P9078-38H; US Biologicals, Salem, MA, USA), anti-PSMB10 (MECL1) (NBP1-58937; Novus Biologicals, Centennial, CO, USA), anti-PSMB6 (E1K9O) (13267S; Cell Signaling Technology, Danvers, MA, USA), anti-PSMB7 (E1L5H) (13207S; Cell Signaling Technology), and anti-PSMB5 (ab3330; Abcam). β-actin (A5316; Sigma-Aldrich) levels were assessed as a loading control. The horseradish peroxidase-conjugated IgG secondary antibody (1:10,000 dilution; Life Technologies Inc.) was used for chemiluminescent signal detection using the ImageQuant LAS 4000 system (GE Healthcare Life Sciences, Marlborough, MA, USA).

### 2.8. Humanized SUM-149 PT Xenograft Mouse Model

The impact of M3258 treatment on the TME of TNBC/IBC was evaluated using SUM-149 PT cells xenografted to humanized mice. For humanization, 4-week-old female NOD.Cg-Prkdcscid Il2rgtm1Wjl Tg(CMV-IL3,CSF2,KITLG)1Eav/MloySzJ mice (Jackson Laboratory, Bar Harbor, ME, USA) were irradiated at 150 centigray and then injected with human CD34^+^ hematopoietic stem cells (3.5 × 10^4^/mouse; Stemcell Technologies, MA, USA) via the tail vein. At week 12 after stem cell inoculation, human CD45^+^ white blood cell (hCD45^+^) engraftment was confirmed by flow cytometry. Mice with more than 25% hCD45^+^ cells in total peripheral blood mononuclear cells were considered successfully humanized and were engrafted in a single mammary fat pad with SUM-149 PT cells (4 × 10^6^) resuspended in 50% Matrigel. Once tumors had reached a volume of 75 to 150 mm^3^, mice (7/group) were treated orally every day with either vehicle or M3258 (10 mg/kg) for 13 days. Tumor volume and mouse body weight were measured twice per week. Tumor volume (mm^3^) was calculated using the formula V = (L × W^2^)/2, in which V, W, and L represent tumor volume, width, and length, respectively. At the end of the study, tumor samples were collected and processed for gene expression analysis (see Section 2.9 below). All animals were maintained and handled in accordance with the guidelines of the MD Anderson Institutional Animal Care and Use Committee (ACUF #00001430-RN02).

### 2.9. Single-Cell RNA Sequencing (scRNA-seq) from SUM-149 PT Tumors

Data from scRNA-seq were demultiplexed and pre-processed using Cell Ranger version 3.1 (10X Genomics, Pleasanton, CA, USA). High-dimensional analyses were performed using SNN with Seurat version 3 [34,35], followed by Uniform Manifold Approximation and Projection dimensional reduction [36]. The top variant genes in each cell cluster were checked and assigned to the corresponding cell types. The FindMarkers function in Seurat was used to identify the top variant genes in each cell cluster, and the top 10 genes were used for comparison. The variant genes expressed in at least 25% of cells in a cluster were selected. Cells exhibiting expression of at least 500 transcripts were used for scRNA-seq analysis after pooling of counts across each sample. Cell-type-specific differential gene expression (DGE) analysis of T cells was performed with Seurat version 3. Pathway analysis was performed with R package fgsea [37] using a Gene Matrix Transposed file converted from human MSigDB. Immune cells were further classified by specific markers for T cells, dendritic cells (FSCN1^+^), mast cells (MS4A2^+^/FCER1^+^), and macrophages. T cells were further classified as CD4^+^ T cells, CD8^+^ T cells (CD8A^+^/CD8B^+^), and T regulatory cells (Tregs, FOXP3^+^), and macrophages were classified as being of M1 phenotype (CD68^+^/CD80^+^/CD86^+^) or M2 phenotype (CD68^+^/CD206^+^/CD163^+^). DGE analysis was performed between the vehicle-treated and M3258-treated samples using DESeq2 after normalization of the count data. *p* values were adjusted for multiple testing with the Benjamini–Hochberg method [38]. Gene set enrichment analysis was performed using the Hallmark and KEGG Pathway databases and specific gene sets.

### 2.10. Flow Cytometry Analysis of THP-1 Cell–Derived M1 and M2 Macrophages

THP-1 cells were seeded at 5 × 10^5^ cells/well in 6-well plates and cultured in the presence of 320 nM phorbol 12-myristate 13-acetate (PMA, Sigma-Aldrich) for 24 h at 37 °C. The cells were then pre-treated with M3258 (0.5 µM or 5 µM) for 2 h and then differentiated into either M1 macrophages by stimulation with recombinant human IFNγ (20 ng/mL; PeproTech Inc., Rocky Hill, NJ, USA) and lipopolysaccharide (50 ng/mL; Santa Cruz Biotechnology, Dallas, TX, USA) or M2 macrophages by stimulation with interleukin-4 (IL-4, 20 ng/mL; PeproTech Inc.) and IL-13 (20 ng/mL; PeproTech Inc.) for 48 h at 37 °C. The cells were then collected and incubated with anti-CD68-APC, anti-CD80-FITC, and anti-CD206-PE antibodies (all from BioLegend, San Diego, CA, USA) at 4 °C for 30 min prior to flow cytometry analysis using a Thermo Fisher Attune NxT BRYV6 device (Thermo Fisher Scientific Inc.).

### 2.11. In Vitro Cell Migration and Invasion Assays Under Co-Culture Conditions

Migration assays were performed using 24-well micro-chemotaxis chambers (Corning Inc., Corning, NY, USA). THP-1 cells (5 × 10^4^/well) were seeded in 24-well micro-chemotaxis chambers and differentiated into M1 or M2 macrophages using the stimulation protocols described in Section 2.10 above. Following washes with PBS, 750 µL of medium supplemented with 10% FBS was added to each well. SUM-149 PT and BCX-010 cells (1 × 10^5^/well) resuspended in FBS-free medium were then added to the upper chambers of transwells with 8-µm pores (Corning Inc.), which were then placed in the micro-chemotaxis chambers. The cells were allowed to migrate for 16 to 24 h and then fixed and stained with the Kwik–Diff Stain kit (Thermo Fisher Scientific Inc.).

Invasion assays with SUM-149 PT and BCX-010 cells were performed as described above for migration assays with the following modifications. The upper chambers of transwells were pre-coated with 100 µL of growth factor–reduced Matrigel (Corning Inc.) diluted at 1:10 in FBS-free RPMI 1640 medium, and cells were allowed to invade for 48 h.

Images of migrated or invaded cells were scanned using the PathScan Enabler IV scanner (Meyer Instruments Inc., Houston, TX, USA) and quantified using ImageJ software (https://imagej.net/ij/, 2021, National Institutes of Health, Bethesda, MD, USA). Conditioned media (CM) from M1 and M2 macrophages (differentiated from THP-1 cells as described in Section 2.10 above) were tested in each assay to assess their pro-chemotactic and pro-invasive effects on TNBC/IBC cells.

To assess the impact of M3258 on SUM-149 PT and BCX-010 cell migration and invasion, each cell line was first pre-treated overnight with IFNγ (50 µg/mL), followed by incubation with M3258 at 1 µM or 5 µM for 2 h. The cells were then collected and resuspended in FBS-free RPMI 1460 medium containing M3258 (1 µM or 5 µM) and applied in the migration and invasion assays, as described above.

### 2.12. Statistical Analysis

For digital image analysis of expression levels of target proteins on IHC-stained TNBC tumor samples, tabular digital image analysis readouts were exported from HALO for statistical analysis using R software version 4.2.2. The correlation of LMP7, PD-L1, and CD8 expression in the tumor cell area of human TNBC samples was investigated by means of Pearson’s coefficient. The data were visualized by means of correlation plots, including a linear fit with a 95% confidence band.

For the in vitro and in vivo studies, all data are presented as mean ± standard deviation. GraphPad Prism software version 8 was used for statistical analyses. Differences between 2 groups were analyzed using a 2-tailed Student *t*-test, and differences among multiple groups were analyzed by Sidak multiple comparisons test following 2-way analysis of variance. *p* < 0.05 was considered statistically significant.

## 3. Results

### 3.1. LMP7 Expression Correlates with CD8^+^ T Cell Infiltration and PD-L1 Expression in TNBC

LMP7 has previously been implicated in the tumor infiltration and activation of diverse immune cell types, such as CD8^+^ T cells, across cancer types, including melanoma and breast cancer [31,39,40]. We therefore assessed the relationships between protein expression of LMP7, CD8, and PD-L1 in human TNBC tumors. As a first step, we characterized the specificity of the anti-LMP7 monoclonal antibody clone EPR14482(B) in IHC analyses. Treatment of the human lung cancer cell line A549 in vitro with IFNγ (100 U/mL), a known inducer of LMP7 expression [4], led to an increased intensity of LMP7 staining with EPR14482(B) by IHC (Supplementary Figure S1A). Meanwhile, CRISPR-based genetic ablation of LMP7 (gene name *PSMB8*) in the human pancreatic cancer cell line SW 1990 resulted in complete loss of LMP7 staining with EPR14482(B) (Supplementary Figure S1B). Together, these analyses confirmed the validity of the EPR14482(B) antibody for IHC analysis of LMP7.

**Figure 1:**
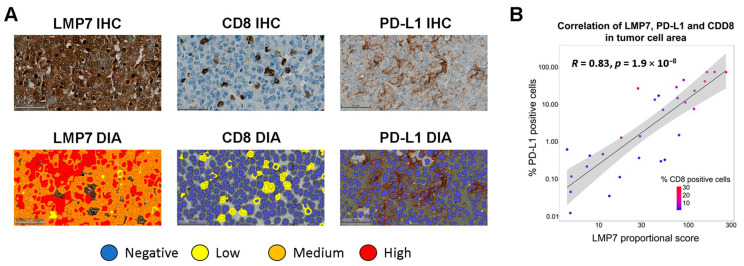
Expression of LMP7, CD8, and PD-L1 in human TNBC samples. (**A**) IHC staining and corresponding digital image analysis (DIA) of LMP7, CD8, and PD-L1 expression in a single TNBC sample (case QP1367) demonstrating high LMP7 expression. (**B**) Correlation of LMP7, CD8, and PD-L1 expression in the tumor cell area of human TNBC samples from the TNBC cohort (*n* = 29).

A unique cohort of 29 human TNBC samples (hereafter referred to as the TNBC cohort) was used to assess the relationships between the protein expression levels of LMP7, CD8, and PD-L1. LMP7 staining intensity differed between individual TNBC tumor samples (Figure 1A,B and Supplementary Figure S2), consistent with previous reports demonstrating elevated IP gene expression in only a subset of TNBC patients [30,31]. In the TNBC cohort, a strong correlation was observed between LMP7 expression, percentage CD8^+^ T cells, and percentage PD-L1^+^ tumor cells (*R* = 0.83, *p* = 1.9 × 10^−8^; Figure 1B).

To corroborate these findings, we assessed the correlation between gene expression of LMP7 (gene name *PSMB8*) and PD-L1 (gene name *CD274*) and signatures for CD8^+^ T cells and IFN signaling in the TNBC cohort (25 samples were available for RNAseq analysis, Supplementary Figure S3) and the TNBC cohort of The Cancer Genome Atlas (TCGA, hereafter referred to as the TCGA TNBC cohort, *n* = 123, Supplementary Figure S4). LMP7 gene expression significantly correlated with the CD8^+^ T cell signature (*R* = 0.8, *p* = 1.6 × 10^−6^ in the TNBC cohort; *R* = 0.59, *p* = 5.1 × 10^−13^ in the TCGA TNBC cohort), IFN signature (*R* = 0.85, *p* = 5.7 × 10^−8^ in the TNBC cohort; *R* = 0.69, *p* < 2.2 × 10^−16^ in the TCGA TNBC cohort), and PD-L1 gene expression (*R* = 0.78, *p* = 3.4 × 10^−6^ in the TNBC cohort; *R* = 0.63, *p* = 8.9 × 10^−15^ in the TCGA TNBC cohort) (Supplementary Figures S3 and S4). Median LMP7 gene expression was significantly higher in the TCGA TNBC cohort compared to that in normal human breast tissue (*p* = 5.7 × 10^−10^; Supplementary Figure S5). This strong correlation between LMP7 expression and CD8^+^ T cell abundance, IFN signaling, and PD-L1 expression suggests that LMP7 may play a role in shaping the CD8^+^ T cell-related inflammatory makeup of TNBC.

### 3.2. Effects of IFNγ and M3258 on Expression of IP and Constitutive Proteasome Subunits

**Figure 2:**
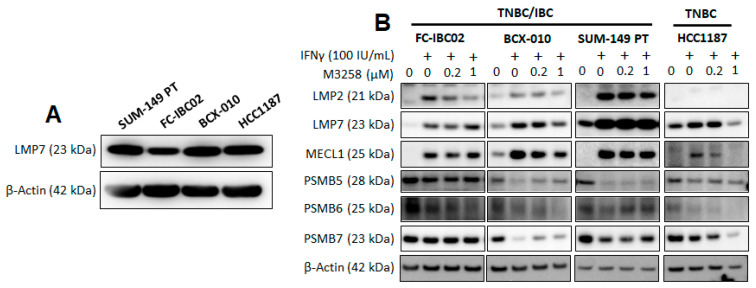
Effects of M3258 and IFNγ on the expression of IP and constitutive proteasome subunits in TNBC/IBC cells. (**A**) Western blot analysis of the basal expression levels of LMP7 in TNBC/IBC cell lines. Cells were cultured for 48 h at 37 °C before proteins were extracted and analyzed. (**B**) Western blot analysis of the effects of M3258 on the expression of IP and constitutive proteasome subunits in TNBC/IBC cells. After overnight incubation in the presence or absence of recombinant human IFNγ (100 IU/mL), the cells were treated with M3258 at the indicated concentrations for 48 h at 37 °C. Proteins were then extracted and analyzed. β-actin levels in the protein extracts were assessed to control for equivalent protein loading in each gel lane. Original western blots are presented in File S1.

The basal expression of LMP7 was assessed in a panel of TNBC/IBC cell lines in vitro. Basal LMP7 expression was detectable in the TNBC/IBC cell lines SUM-149 PT, FC-IBC02, and BCX-010, as well as the TNBC cell line HCC1187 (Figure 2A). We next examined the effects of M3258 treatment on the expression of the IP subunits LMP2, LMP7, and MECL1 and the constitutive proteasome subunits PSMB5 (β1), PSMB6 (β2), and PSMB7 (β5) by Western blotting. Given the correlation between LMP7 and IFN signaling in human TNBC samples, as shown above (Figure 1B), we pre-stimulated each cell line overnight with or without IFNγ (100 IU/mL). In the TNBC/IBC cell lines FC-IBC02, BCX-010, and SUM-149 PT, IFNγ led to a pronounced increase in the expression of all three IP subunits (Figure 2B). In the TNBC cell line HCC1187, only LMP7 and MECL1 were induced by IFNγ; LMP2 was undetectable. IFNγ treatment decreased the expression of one or more constitutive proteasome subunits in all cell lines with the exception of FC-IBC02. These observations suggest that IFNγ skews the proteasome composition of TNBC/IBC cells towards dominant IP expression.

**Figure 3:**
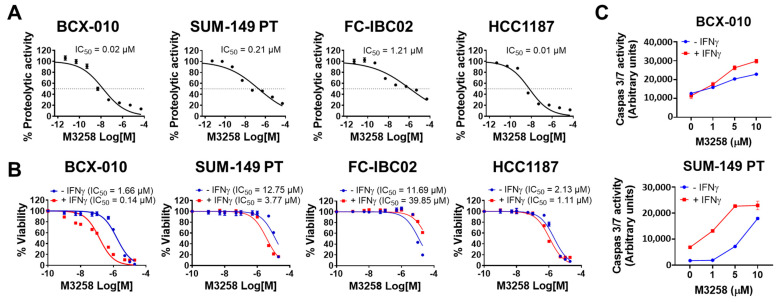
M3258 suppressed LMP7 proteolytic activity, reduced viability, and induced apoptosis in TNBC/IBC cells. (**A**) Inhibition of LMP7 proteolytic activity in the indicated TNBC/IBC cell lines. Cells were treated with M3258 for 2 h before assessment of LMP7 activity. Results are presented as mean ± standard deviation from triplicate samples, and IC_50_ values are indicated. (**B**) Inhibition of viability of the indicated TNBC/IBC cell lines by M3258. Cells were pre-treated with human IFNγ (100 IU/mL) overnight prior to the addition of M3258 (0 to 50 µM) for 72 h. Cell viability was measured using the CellTiter-Blue assay. Results are presented as mean ± standard deviation from triplicate samples, and IC_50_ values are indicated. (**C**) Induction of apoptosis in the indicated TNBC/IBC cell lines by M3258. Cells were pre-treated with IFNγ (25 IU/mL) prior to the addition of M3258 at the indicated concentrations for 24 h. Apoptosis was measured using the Caspase-Glo 3/7 assay. Results are presented as mean ± standard deviation from triplicate samples.

Treatment with the LMP7 inhibitor M3258 at 0.2 µM for 48 h did not have a pronounced effect on the expression of any of the IP or constitutive proteasome subunits, compared to IFNγ alone. A decrease in the expression of selected IP and/or constitutive proteasome subunits was discernible in some cell lines treated with M3258 at 1 µM. This observation is unlikely to be due to off-target cytotoxic effects (see characterization of M3258 cytotoxicity in Figure 3) and may reflect adaptive changes in proteasome subunit expression, as has previously been described [13,41].

### 3.3. Suppression of LMP7 Activity by M3258 Reduces Viability and Induces Apoptosis in TNBC/IBC Cells In Vitro

We evaluated the effect of M3258 on LMP7 proteolytic activity in TNBC/IBC cell lines in vitro. M3258 effectively suppressed LMP7 activity in the TNBC/IBC cell lines BCX-010 (IC_50_ = 0.02 µM), SUM-149 PT (IC_50_ = 0.21 µM), and FC-IBC02 (IC_50_ = 1.21 µM), as well as the TNBC cell line HCC1187 (IC_50_ = 0.01 µM) (Figure 3A).

We next tested the effect of M3258 on the viability of the same panel of cells. M3258 reduced the viability of BCX-010, SUM-149 PT, FC-IBC02, and HCC1187 cells in a dose-dependent manner, with IC_50_ values greater than 1 µM (Figure 3B). Pre-stimulation with IFNγ (100 IU/mL) increased the potency of M3258 in the BCX-010 (11.9-fold), SUM-149 PT (3.4-fold), and HCC1187 (1.9-fold) cell lines and reduced the potency of M3258 (3.4-fold) in FC-IBC02.

To determine whether M3258 reduced TNBC/IBC cell viability by inducing apoptosis, we treated BCX-010 and SUM-149 PT cells with M3258 for 24 h and measured caspase 3/7 activity. M3258 treatment increased caspase 3/7 activity in a dose-dependent manner in both cell lines (Figure 3C). IFNγ pre-treatment potentiated the induction of caspase 3/7 activity by M3258 in each cell line. Together, these findings indicate an intrinsic dependency of TNBC/IBC cells on LMP7 function, particularly when LMP7 expression is induced by IFNγ.

### 3.4. M3258 Inhibits Tumor Growth in a SUM-149 PT Xenograft Model In Vivo

**Figure 4:**
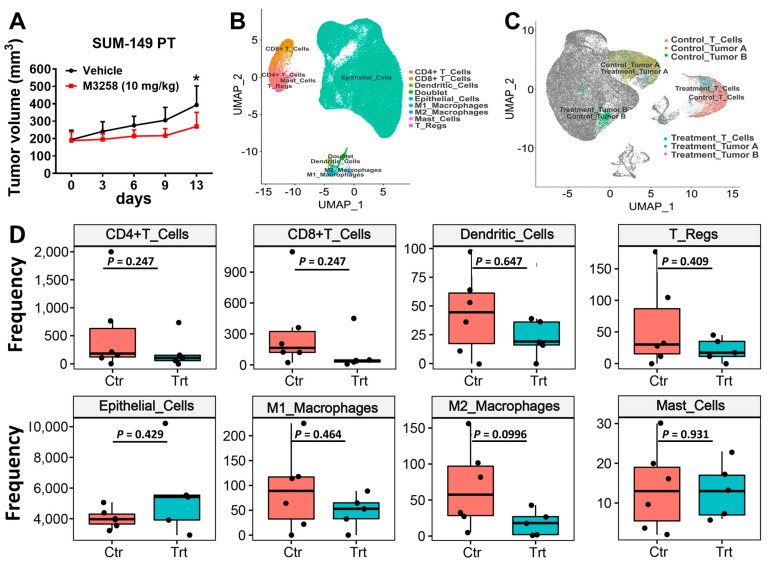
Impact of M3258 on tumor growth and immune cell clusters in the SUM-149 PT xenograft model in humanized mice. (**A**) SUM-149 PT tumors were established in humanized mice as described in Materials and Methods Section 2.8. Mice were treated with vehicle or M3258 (10 mg/kg) orally once per day for 13 days. Mean tumor volume ± standard deviation is shown for each treatment group; * *p* < 0.01, determined by Sidak multiple comparisons test following 2-way analysis of variance. (**B**) Clusters of cell populations from terminal SUM-149 PT xenograft tumors from the experiment indicated in A, as analyzed by scRNA-seq. SUM-149 PT xenografts were divided into 9 clusters, including 1 doublet cell population and 8 singlet cell populations: epithelial cells (tumor cells), CD4^+^ T cells, CD8^+^ T cells, Tregs, M1 macrophages, M2 macrophages, dendritic cells, and mast cells. (**C**) The SUM-149 PT tumor cells were further divided into tumor compartments A and B. (**D**) Effect of M3258 on immune cell composition of SUM-149 PT tumors. Data are presented as mean ± standard deviation and *p* values are indicated. Abbreviations: Ctr, vehicle control; Trt, M3258-treated.

To confirm our in vitro findings, we conducted an in vivo study to assess the antitumor efficacy of M3258 using a humanized SUM-149 PT xenograft mouse model. M3258 administered orally at 10 mg/kg once per day significantly inhibited the growth of SUM-149 PT xenograft tumors compared to the growth in vehicle-treated mice (*n* = 7; *p* < 0.01 on day 13; Figure 4A). No significant differences in body weight were observed between the vehicle-treated and M3258-treated mice, indicating that M3258 treatment was tolerated (Supplementary Figure S6).

### 3.5. Effects of M3258 on Immune Cell Composition of SUM-149 PT Tumors

To examine the impact of M3258 treatment on the TME, we performed sc-RNAseq analysis of the SUM-149 PT xenograft tumors from the experiment depicted in Figure 4A. On the basis of DGE profiles, the tumors were divided into nine clusters, including one doublet cell population and eight singlet cell populations representing epithelial cells (tumor cells), CD4^+^ T cells, CD8^+^ T cells, Tregs, M1 macrophages, M2 macrophages, dendritic cells, and mast cells (Figure 4B). Epithelial cells were further divided into tumor A and tumor B clusters on the basis of their DGE profiles (Figure 4C). The abundance of CD4^+^ T cells, CD8^+^ T cells, Tregs, dendritic cells, epithelial cells, M1 macrophages, M2 macrophages, and mast cells was not significantly different in tumors from M3258-treated and vehicle-treated mice (Figure 4D). The most pronounced trend observed following M3258 treatment was a reduction in M2 macrophages (*p* = 0.0996); less prominent reductions were observed for tumor abundance of CD4^+^ T cells (*p* = 0.247) and CD8^+^ T cells (*p* = 0.247). The lack of statistical significance may be due to the relatively small size of each treatment group (*n* = 7) and differences in immune compositions between individual mice due to varying degrees of humanization. Future studies using larger data sets and utilizing orthogonal methodologies (e.g., scRNA-seq, IHC, and flow cytometry) are warranted to further dissect the effects of M3258 on the immune cell architecture of TNBC/IBC tumors.

### 3.6. Tumor and Immune Cell Pathways Affected by M3258 Treatment in SUM-149 PT Tumors

**Figure 5:**
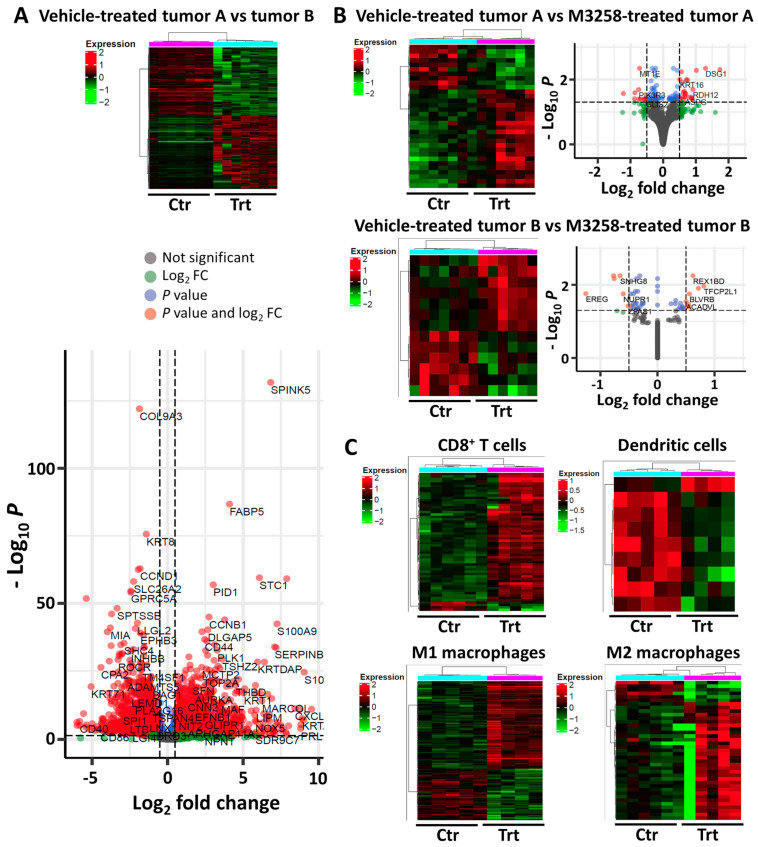
DGE analysis in tumor and immune compartments of SUM-149 PT tumors from humanized mice treated with vehicle or M3258. Single-cell sequencing data from Figure 4 were used to identify differentially expressed genes. Heatmaps were generated to illustrate significant DGEs (FDR −0.05, Log_2_ fold change cutoff −0.5) from these comparisons for different tumor and cell types. (**A**) Heatmaps and volcano plots show the patterns of DGE in tumor A and tumor B compartments from mice treated with vehicle. (**B**) Heatmaps and volcano plots show the patterns of DGE in tumor A (left) and tumor B (right) compartments from mice treated with vehicle versus M3258. (**C**) Heatmaps show the patterns of DGE in CD8^+^ T cells, dendritic cells, M1 macrophages, and M2 macrophages in tumors from mice treated with vehicle versus M3258. Abbreviations: Ctr, vehicle control; Trt, M3258-treated.

Tumor compartments A and B exhibited different DGE profiles (Figure 5A), confirming the cellular heterogenicity of the SUM-149 PT tumors. In gene set enrichment analysis using the Hallmark and KEGG databases, tumor compartment A was found to be enriched for pathways associated with an aggressive tumor phenotype, such as epithelial to mesenchymal transition, cell cycle progression (e.g., E2F targets, G2M checkpoint, MYC targets, and mitotic spindle), angiogenesis, cell stress (e.g., unfolded protein response, hypoxia, and cytosolic DNA sensing), TNFα-related inflammation, and the KRAS and Hedgehog pathways (Supplementary Tables S1 and S2), while tumor compartment B was found to be enriched for pathways associated with IFNα and IFNγ signaling, cell adhesion, antigen processing and presentation, and phagosome activity (Supplementary Tables S3 and S4).

A pronounced change in DGE profiles in tumor compartments A and B was observed following M3258 treatment (Figure 5B and Supplementary Tables S5 and S6). Consistent with the reduced growth of SUM-149 PT tumors under M3258 treatment described in Figure 4A above, M3258 suppressed pathways associated with cell proliferation (e.g., MYC targets, G2M checkpoint, E2F targets, WNT, and ERBB), protein expression and homeostasis (e.g., ribosome activity and antigen processing and presentation), and cell motility (e.g., cell adhesion) (Supplementary Tables S7 and S8). M3258 also suppressed inflammation-related pathways (e.g., TNFα, IFNγ, IL-2/STAT5, and JAK/STAT) (Supplementary Tables S7 and S8).

As shown in Figure 5C, M3258 treatment led to substantial changes in DGE profiles in CD8^+^ T cells (Supplementary Table S9), dendritic cells (Supplementary Table S10), M1 macrophages (Supplementary Table S11), and M2 macrophages (Supplementary Table S12). Pathway analysis indicated suppression of the IFNα response in CD8^+^ T cells following M3258 treatment (Supplementary Table S13). In M1 macrophages, M3258 suppressed the TNFα signaling pathway and enriched the proteasome pathway (Supplementary Tables S13 and S14). Consistent with the trend of reduced M2 macrophage abundance shown in Figure 4D, in M2 macrophages, M3258 suppressed pathways related to cell proliferation (E2F targets, G2M checkpoint, and MYC targets). M3258 suppressed the ribosome pathway in CD8^+^ T cells, M1 and M2 macrophages, and dendritic cells, suggesting potential disruption of protein homeostasis. Given the critical role of CD8^+^ T cells in tumor surveillance and immunotherapy response [42], we examined further gene expression patterns related to the activation status of these cells and other T cell subsets. In CD8^+^ T cells, M3258 treatment enriched gene expression indicative of CD8^+^ T cell activation (Supplementary Figure S7), including *UCHL1* [43,44] (Supplementary Table S15). In contrast, no significant effect of M3258 was detected on CD8^+^ T cell exhaustion or the activation of CD4^+^ T cells and Tregs (Supplementary Figure S7).

### 3.7. M3258 Enhances THP-1 Differentiation toward M1 Macrophages In Vitro

**Figure 6:**
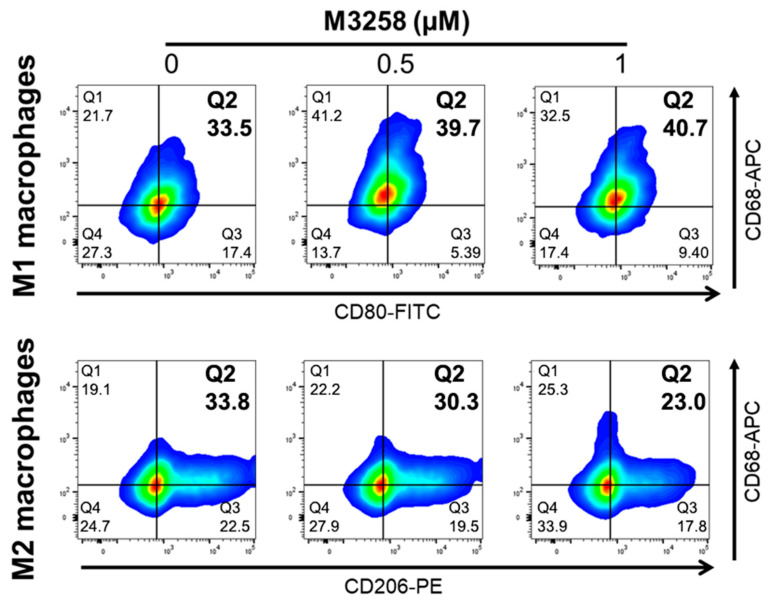
Representative images showing that M3258 promoted THP-1 monocyte differentiation into M1 macrophages while suppressing M2 differentiation. PMA-primed THP-1 cells were pre-treated with M3258 (0.5 µM or 1 µM) for 2 h and then differentiated into M1 or M2 macrophages. After 48 h, cells were collected, stained with anti-CD68-APC, anti-CD80-FITC, and anti-CD206-PE antibodies, and analyzed by flow cytometry. Experiments were performed individually 3 times.

Since M2 macrophage abundance decreased following M3258 treatment in SUM-149 PT tumors (Figure 4D), we further investigated the effect of M3258 on M1 and M2 macrophage differentiation. Flow cytometry analysis showed that 48-h treatment with M3258 (0.5 or 1 µM) promoted THP-1 monocyte differentiation toward M1 macrophages (Figure 6, top panel) while reducing differentiation toward M2 macrophages (Figure 6, bottom panel). These findings suggest that M3258 skews THP-1 differentiation toward an M1 macrophage phenotype.

### 3.8. M3258 Inhibits M2 Macrophage-Induced TNBC/IBC Cell Migration and Invasion In Vitro

**Figure 7:**
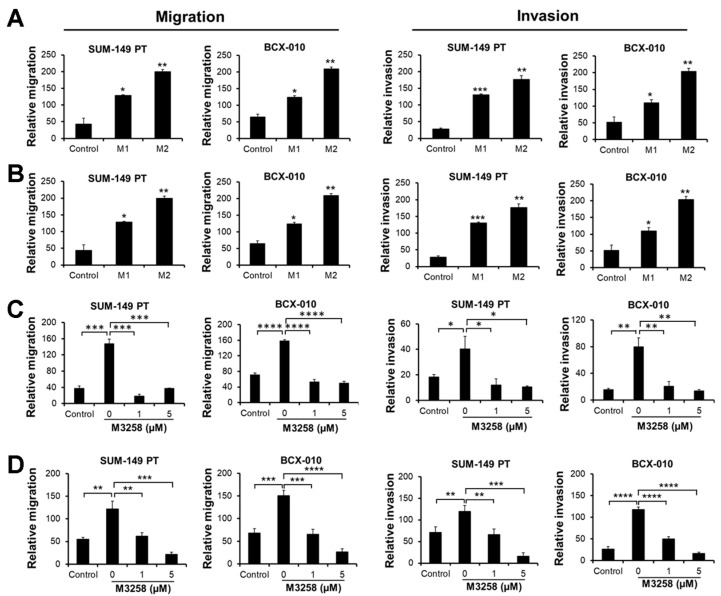
M3258 suppressed macrophage-induced migration and invasion of TNBC/IBC cells in vitro. (**A**) The migration and invasion of SUM-149 PT and BCX-010 cells towards M1 or M2 macrophages derived from differentiated THP-1 cells was assessed in vitro using micro-chemotaxis chambers. (**B**) SUM-149 PT and BCX-010 cell migration and invasion toward CM from M1 or M2 macrophages were assessed as described in (**A**). (**C**) Effect of M3258 treatment on M2-macrophage-induced migration and invasion of SUM-149 PT and BCX-010 cells in vitro. Cells were pre-treated with IFNγ (50 µg/mL) and then resuspended in FBS-free RPMI 1460 medium containing M3258 (1 µM or 5 µM) prior to use in assays to assess migration and invasion toward M2 macrophages, as described in (**A**). (**D**) The effect of M3258 on the migration and invasion of SUM-149 PT and BCX-010 cells toward M2 macrophage CM was assessed as described in (**C**). * *p* < 0.05, ** *p* < 0.01, *** *p* < 0.001, **** *p* < 0.0001, determined by a 2-tailed Student *t*-test.

Given the established role of macrophages in tumor progression [45], we assessed the effect of macrophages on TNBC/IBC cells using migration and invasion assays. Indirect co-culture with M1 or M2 macrophages significantly enhanced the transwell migration and invasion of SUM-149 PT and BCX-010 cells (Figure 7A), and M2 macrophages exhibited a more pronounced effect.

Additionally, CM from M1 or M2 macrophages induced the migration and invasion of SUM-149 PT and BCX-010 cells (Figure 7B), with M2 macrophage CM demonstrating greater activity. These data indicate that paracrine factors produced by M2 macrophages are potent inducers of migration and invasion of TNBC/IBC cells. Treatment of SUM-149 PT and BCX-010 cells with M3258 at 1 or 5 µM significantly reduced their migration and invasion towards M2 macrophages and M2 macrophage CM (Figure 7C,D). Together, these findings demonstrate that M3258 effectively inhibits M2 macrophage-primed migration and invasion of TNBC/IBC cells, a mechanism that may contribute to M3258’s observed efficacy in the SUM-149 PT in vivo humanized tumor model described above.

## 4. Discussion

Proteasome inhibition represents a key pillar of therapeutic intervention in MM. Three pan-PIs, bortezomib, carfilzomib, and ixazomib, have been approved by the US Food and Drug Administration for MM treatment [46]. The high utility of pan-PIs in MM stems from the intrinsic dependency of MM cells on components of the ubiquitin–proteasome system, which arises from the aberrant expression of monoclonal immunoglobulin, as well as the role of ubiquitin–proteasome system components in degrading specific tumor suppressors, proapoptotic proteins, and cell-cycle inhibitory proteins [9]. Compared with their activity in MM, pan-PIs have shown less promising activity in other hematolymphoid cancers and solid tumors [47,48]. This less promising activity may be due to the lower ubiquitin–proteasome system dependency of these other cancer cell types and the diverse toxic effects associated with pan-PIs that limit doses to sub-therapeutic levels [10].

Recent studies analyzing large transcriptomics datasets have identified overexpression of IP catalytic subunits, including LMP7, in subsets of patients with several types of solid tumors, including melanoma and breast cancer [30,31,40,49]. These findings have raised significant interest in understanding the potential application of selective IP inhibitors in patients with solid tumors with high IP subunit expression. However, until recently, the lack of highly selective IP subunit inhibitors with properties enabling in vivo application has hampered such investigations. M3258, a highly selective and orally bioavailable LMP7 inhibitor, was recently described to exhibit strong antitumor activity in preclinical models of MM [13,15], as well as a more favorable non-clinical safety profile in rats and dogs than the safety profile described for pan-PIs [14]. In this study, we used M3258 in preclinical models to investigate the therapeutic potential of LMP7 inhibition in TNBC/IBC.

Consistent with previous reports described above [30,31,49], our analysis of TNBC transcriptomics data from TCGA identified a strong correlation between expression of LMP7 (*PSMB8*) and infiltration of CD8^+^ T cells, IFN pathway activation, and expression of PD-L1 (*CD274*). Using a novel panel of TNBC samples, we were able to corroborate these gene expression findings and additionally demonstrate a strong correlation between the protein expression of LMP7, CD8, and PD-L1.

Using in vitro assay systems, we demonstrated that the inhibition of LMP7 activity with M3258 directly suppressed viability and induced apoptosis in several TNBC/IBC cell lines. Consistent with the association between LMP7 and IFN signaling observed in human TNBC samples, IFNγ induced LMP7 expression in these TNBC/IBC cell lines and sensitized selected cell lines to the effects of M3258. In keeping with these findings, M3258 treatment significantly suppressed tumor growth in an in vivo model of TNBC/IBC in humanized mice. Together, these data suggest that the induction of LMP7 expression by IFNγ, potentially derived in vivo from tumor-residing CD8^+^ T cells, establishes an intrinsic dependency of TNBC/IBC cells on LMP7 function.

The rich influx of CD8^+^ T cells, PD-L1 expression, and activated IFN signaling in TNBC patient tumors with high LMP7 expression prompted us to investigate the effects of M3258 on the immune landscape of TNBC/IBC. Our previous investigation using in vitro cultured primary human immune cells indicated that M3258 affected the proliferation of B cells and T cells, but not other cell types such as macrophages [13]. This contrasted with the more pleiotropic cytotoxicity of the pan-PI ixazomib on diverse human cells [50]. In the study reported here, in the in vivo SUM-149 PT model of TNBC/IBC in humanized mice, M3258 treatment did not significantly impact the abundance of CD8^+^ T cells or other immune cells. However, M3258 did lead to CD8^+^ T cell activation, as exemplified by the increased expression of *UCHL1* [43,44]. Analysis of the effect of M3258 on immune cell types showed that the most pronounced, albeit non-significant, effect of M3258 was a decrease in the tumor abundance of M2 macrophages. M2 macrophages have been shown to contribute to the progression of TNBC/IBC in preclinical models [51,52,53]. Using in vitro co-culture systems, we showed that M3258 treatment led to a pronounced suppression of TNBC/IBC cell migration and invasion induced by M2 macrophage-derived soluble mediators. These novel findings suggest the need for future studies to identify the precise M2 macrophage-derived mediators that promote TNBC/IBC cell motility, as well as the molecular mechanisms underpinning the suppressive effects of LMP7 inhibition.

Intriguingly, high IP subunit expression was reported to correlate with better overall prognosis in TNBC [30,31] and improved response to immune checkpoint inhibition in melanoma [40]. This likely reflects the important role of the IP in major histocompatibility complex class I antigen presentation [1] and is consistent with our observation that LMP7 expression correlated with several hallmarks of immune-inflamed tumors, including CD8^+^ T cell infiltration and IFNγ signaling. This raises the potential concern that pharmacological inhibition of the IP may have a detrimental effect on adaptive antitumor immunity and patient outcomes. In contrast to this hypothesis, our study showed that selective LMP7 inhibition with M3258 led to the activation of CD8^+^ T cells in SUM-149 PT TNBC/IBC tumors. Previously published studies investigating the effects of IP inhibitors with different subunit selectivity profiles on antigen presentation and T cell function may provide a potential explanation for this apparent contradiction. Independent groups have demonstrated that inhibition of multiple IP subunits (e.g., dual LMP2/LMP7 inhibition) was required for the effective suppression of antigen presentation and T cell-mediated inflammation in experimental models of autoimmunity, while selective inhibitors of either LMP7 or LMP2 were ineffective [16,54,55]. Based on these findings, it is foreseeable that under conditions of selective LMP7 inhibition in the TME of TNBC/IBC, the activity of the remaining IP subunits LMP2 and MECL1 may be sufficient for the generation of peptides for antigen presentation and activation of CD8^+^ T cells. Suppression of M2 macrophages may provide an additional explanation for the observed activation of CD8^+^ T cells by M3258. M2 macrophages are associated with pro-angiogenic and immunosuppressive functions and display reduced capacity for antigen presentation compared to M1 macrophages [56]. These collective observations suggest that selective LMP7 inhibition may represent a feasible pharmacological approach for reshaping the TME of TNBC/IBC in a manner permissive to CD8^+^ T cell-mediated immune surveillance. Given the critical importance of drug combinations for meaningful efficacy in TNBC, examination of potential synergies between M3258 and orthogonal therapeutic modalities, in particular immunotherapy, will be of high interest for future studies.

## 5. Conclusions

Our study demonstrates for the first time that LMP7 inhibition has potential therapeutic value in LMP7-expressing TNBC/IBC because it directly perturbs cancer cell viability and reshapes the TME, particularly via the activation of CD8^+^ T cells and the suppression of M2 macrophages. These findings provide a basis for further investigation of LMP7 inhibitors like M3258 in solid tumors exhibiting high LMP7 expression, including in combination with agents that modulate the activity of CD8^+^ T cells (e.g., checkpoint inhibitors) and M2 macrophages.

## Data Availability

The datasets generated and/or analyzed in this study are available upon reasonable request.

## Supplementary Materials

The following supporting information can be downloaded at: https://www.mdpi.com/article/10.3390/cancers17111887/s1, Supplementary Materials and Methods: Generation of LMP7 knockout SW 1990 cell lines; Immunohistochemistry (IHC) staining of human cell lines; Gene expression analyses. Supplementary Figures: Supplementary Figure S1. Characterization of the specificity of the rabbit anti-human LMP7 monoclonal antibody clone EPR14482(B)) using human cell lines; Supplementary Figure S2. Immunohistochemistry (IHC) staining and digital image analysis (DIA) of differential LMP7 expression in TNBC samples; Supplementary Figure S3. Correlation of gene expression of LMP7 with signatures for CD8^+^ T cells and IFN, and gene expression of PD-L1 in the TNBC cohort; Supplementary Figure S4. Correlation of gene expression of LMP7 with signatures for CD8^+^ T cells and IFN, and gene expression of PD-L1 in the TCGA TNBC cohort; Supplementary Figure S5. Comparison of LMP7 (PSMB8) gene expression between TCGA normal human breast tissue samples and the TCGA TNBC cohort tumor samples; Supplementary Figure S6. Effect of M3258 treatment on mouse body weight; Supplementary Figure S7. Enrichment of pathways regulating activation and exhaustion of CD8^+^ T cells, as well as activation of CD4^+^ T cells and Tregs, from SUM-149 PT tumors from mice treated with M3258 and vehicle-treated mice. File S1: Full Western blot images. Supplementary Tables: Supplementary Table S1. Pathways enriched in the vehicle-treated tumor compartment A versus tumor compartment B (Hallmark); Supplementary Table S2. Pathways enriched in the vehicle-treated tumor compartment A versus tumor compartment B (KEGG); Supplementary Table S3. Pathways enriched in the vehicle-treated tumor compartment B versus tumor compartment A (Hallmark); Supplementary Table S4. Pathways enriched in the vehicle-treated tumor compartment B versus tumor compartment A (KEGG); Supplementary Table S5. Genes affected by M3258 treatment versus vehicle treatment in tumor compartment A; Supplementary Table S6. Genes affected by M3258 treatment versus vehicle treatment in tumor compartment B; Supplementary Table S7. Major pathways affected by M3258 treatment versus vehicle treatment in tumors (Hallmark); Supplementary Table S8. Major pathways affected by M3258 treatment versus vehicle treatment in tumors (KEGG); Supplementary Table S9. Genes affected by M3258 treatment versus vehicle treatment in CD8^+^ T cells; Supplementary Table S10. Genes affected by M3258 treatment versus vehicle treatment in dendritic cells; Supplementary Table S11. Genes affected by M3258 treatment versus vehicle treatment in M1 macrophages; Supplementary Table S12. Genes affected by M3258 treatment versus vehicle treatment in M2 macrophages; Supplementary Table S13. Major pathways affected by M3258 treatment versus vehicle treatment in immune cells (Hallmark); Supplementary Table S14. Major pathways affected by M3258 treatment versus vehicle treatment in immune cells (KEGG); Supplementary Table S15. Genes affected by M3258 treatment versus vehicle treatment in CD8^+^ T cells.
