# Supplementary material for: LMP7-Specific Inhibitor M3258 Modulates the Tumor Microenvironment of Triple-Negative Breast Cancer and Inflammatory Breast Cancer
Source: Cancers (Basel). 2025 Jun 4;17(11):1887. doi: 10.3390/cancers17111887 (PMC12153562; doi:10.3390/cancers17111887)

Full Western blot images

Full images for LMP7 and β-Actin Western blotting data corresponding to Figure 2A

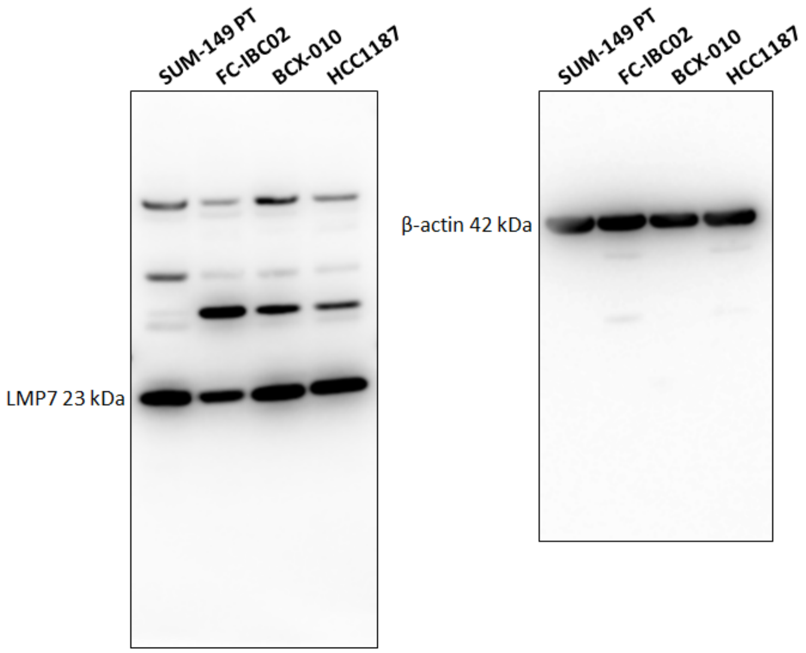

Full images for LMP2, LMP7, and MECL1 Western blotting data corresponding to Figure 2B

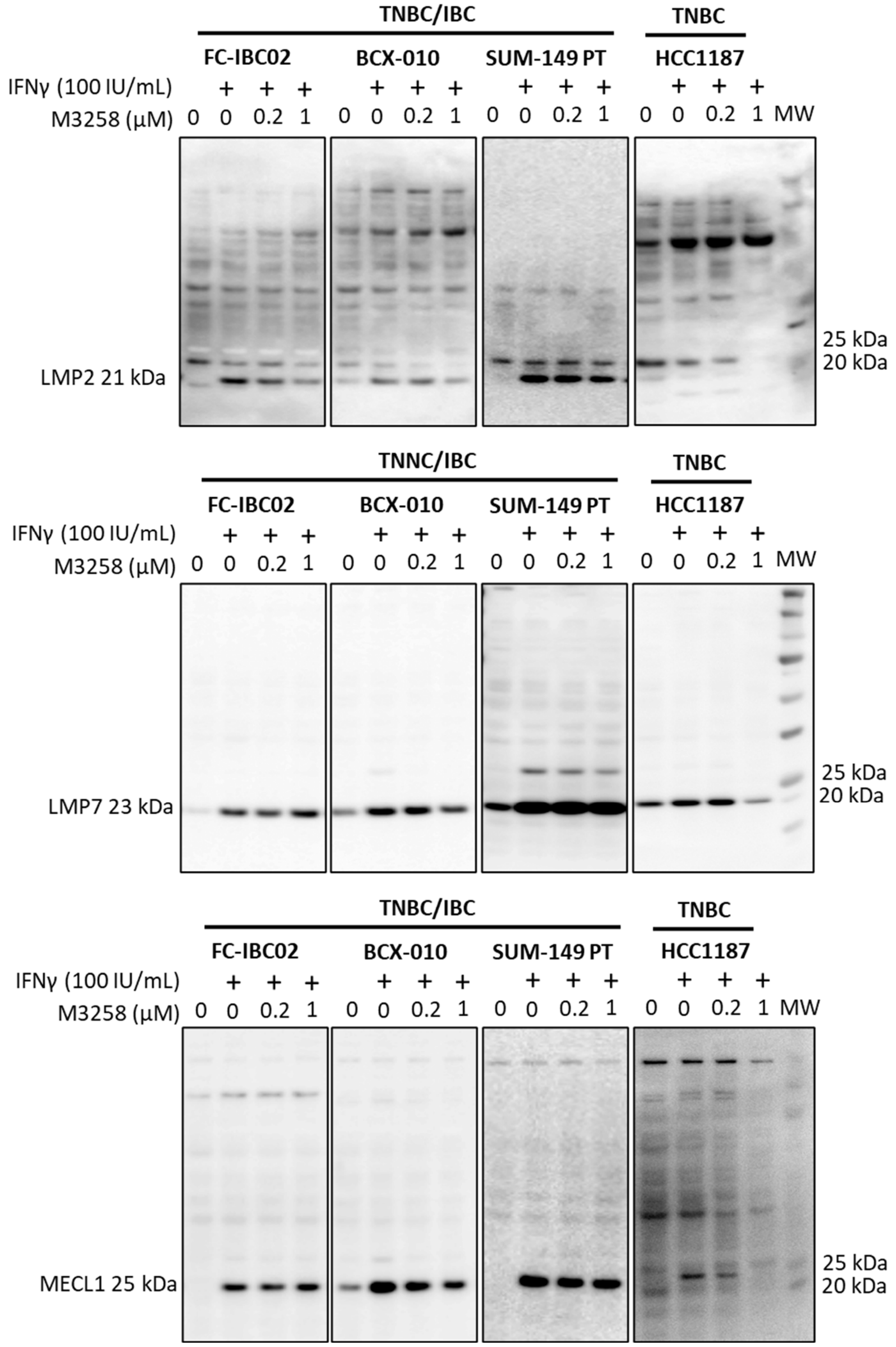

Full images for PSMB5, PSMB6, PSMB7 and β-Actin Western blotting data corresponding to Figure 2B

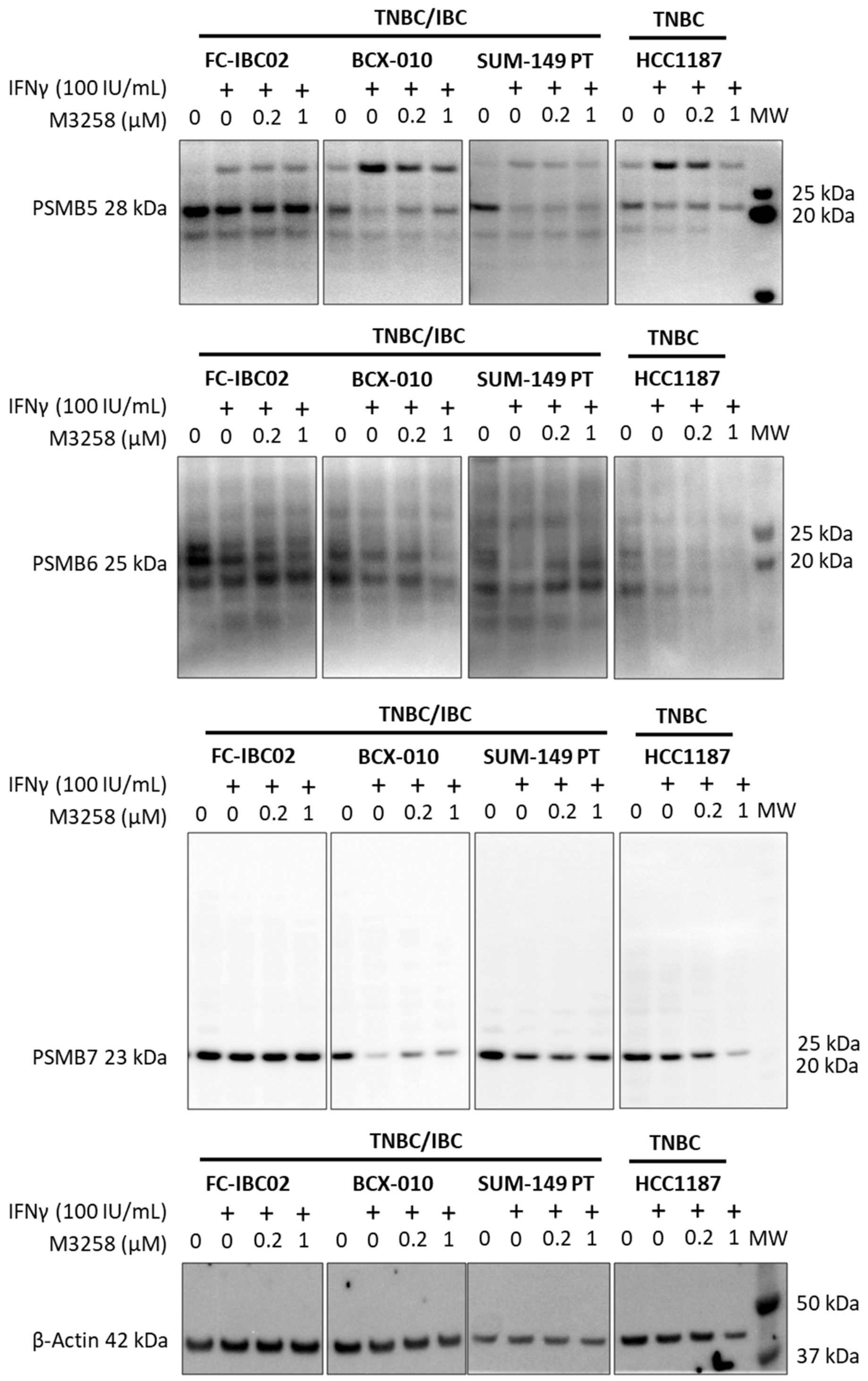

Supplement: Supplementary file 1 [file cancers-17-01887-s001.zip › cancers-3645380-File S1.pdf]
