# Supplementary material for: LMP7-Specific Inhibitor M3258 Modulates the Tumor Microenvironment of Triple-Negative Breast Cancer and Inflammatory Breast Cancer
Source: Cancers (Basel). 2025 Jun 4;17(11):1887. doi: 10.3390/cancers17111887 (PMC12153562; doi:10.3390/cancers17111887)
Supplement: Supplementary file 1 [file cancers-17-01887-s001.zip › cancers-3645380-supplementary.pdf]

LMP7-Specific Inhibitor M3258 Modulates the Tumor Microenvironment of Triple-Negative Breast Cancer and Inflammatory Breast Cancer

Supplementary Materials and Methods

Generation of LMP7 knockout SW 1990 cell lines

LMP7 (gene name *PSMB8*) knock-out clones were generated by Synthego Corporation (Redwood City, CA, USA) using the parental pancreatic cancer cell line SW 1990 and synthetic guideRNAs.

Immunohistochemistry (IHC) staining of human cell lines

IHC staining of LMP7 was performed to determine the binding specificity of the anti-LMP7 antibody (clone EPR14482(B), #ab18060; Abcam, Cambridge, United Kingdom) using human cell lines. In brief, A549 cells (ATCC) were cultured in DMEM medium with 10% fetal calf serum (FCS; Merck Millipore, Burlington, MA, USA) for 24 h followed by treatment with or without 100 U/mL of recombinant human IFNγ (R&D Systems Minneapolis, MN, USA) for an additional 24 h. SW 1990 cells and the LMP7 knockout subclones 1G7 and 3E10 were cultured in DMEM medium with 10% FCS for 24 h. Thereafter, medium was removed and the cells were washed with D-PBS (Invitrogen, Waltham, MA, USA). The cells were then removed from the flask by trypsinization. Between 1 to 2×10^7 cells were resuspended in ice-cold 4% formaldehyde solution (VWR, Radnor, PA, USA) and incubated for 2 h. The cells were then pelleted by centrifugation and resuspended in 30 to 100 µL formaldehyde at 65°C. An equal volume of warm 5% SeaPlaque® Agarose (Lonza, Basel, Switzerland) was then added. The cell-agarose mixtures were then suspended into the wells of a 96-well plate and hardened at room temperature. The cell agarose pellets were then placed into cassettes and formaldehyde-fixed and paraffin-embedded (FFPE) using an automated paraffin embedder (#ASP300S, Leica Biosystems, Deer Park, IL, USA). FFPE cell pellets were sectioned using a microtome (Leica RM 2255, Leica Biosystems) and mounted on Matsunami TOMO® hydrophilic adhesion slides (Matsunami Glass Ind.,Ltd., Osaka, Japan). The immunohistochemical staining procedure was performed on a Discovery® XT (Ventana Medical Systems Inc., Tucson, AZ, USA). After deparaffinization, the sections were heated for epitope retrieval in Tris-EDTA buffer pH 8 (Roche Diagnostics Deutschland GmbH, Germany). Sections were then incubated with 0.5 µg/mL LMP7 antibody and anti-rabbit IgG from the DISCOVERY OmniMap anti-rabbit HRP kit (Roche Diagnostics Deutschland GmbH) and stained with DISCOVERY ChromoMap DAB Kit (Roche Diagnostics Deutschland GmbH). Sections were counterstained with Hematoxylin II (Roche Diagnostics Deutschland GmbH). The slides were then washed in water, dehydrated, and mounted with glass coverslips in permanent mounting media Entellan® Neu (Sigma Aldrich, Germany). Slides were scanned using the NanoZoomer S210 (Hamamatsu Photonics Deutschland GmbH, Germany).

Gene expression analyses

RNA sequencing (RNA-seq) data were used to correlate LMP7 (gene name *PSMB8*) gene expression with other genes using both the TCGA TNBC cohort and the TNBC cohort datasets. The TCGA TNBC RNA-seq datasets (Version 2020-07-23), including datasets of clinical information and gene expression, were downloaded from UCSC Xena via R package UCSCXenaTools. Transcript Per Million (TPM) values were used for each gene, which were then

log-transformed (log2[TPM + 1]). Pearson's correlation coefficients between the expression of genes were calculated based on log-transformed TPM values.

The TNBC cohort RNA-seq datasets were generated at Personalis AG (Switzerland) using the TNBC samples procured from Indivumed GmbH. Gene expression values were quantified using the ACE Cancer Transcriptome Analysis pipeline, which uses STAR version 2.4.2a-p1 to align reads to the National Center of Biotechnology Information hs37d5 annotation 105 reference genome and produces TPM value for each gene. TPM values were log2-transformed for further analysis. Pearson's correlation coefficients between genes were calculated based on log-transformed TPM values.

For comparison of LMP7 (gene name *PSMB8*) gene expression between TNBC and normal human breast tissue, RNA-seq data from the TCGA TNBC cohort and TCGA matching normal human breast samples were analyzed. The TCGA TNBC cohort & Normal RNA-seq datasets (Version 2020-07-23), including datasets of clinical information and gene expression, were downloaded from UCSC Xena via R package UCSCXenaTools. TPM values were used for each gene, which were then log-transformed (log2[TPM + 1]). Pearson's correlation coefficients between the expression of genes were calculated based on log-transformed TPM values. The Wilcoxon Test was used to calculate *P* values for pairwise comparisons.

Supplementary Figures

Supplementary Figure S1

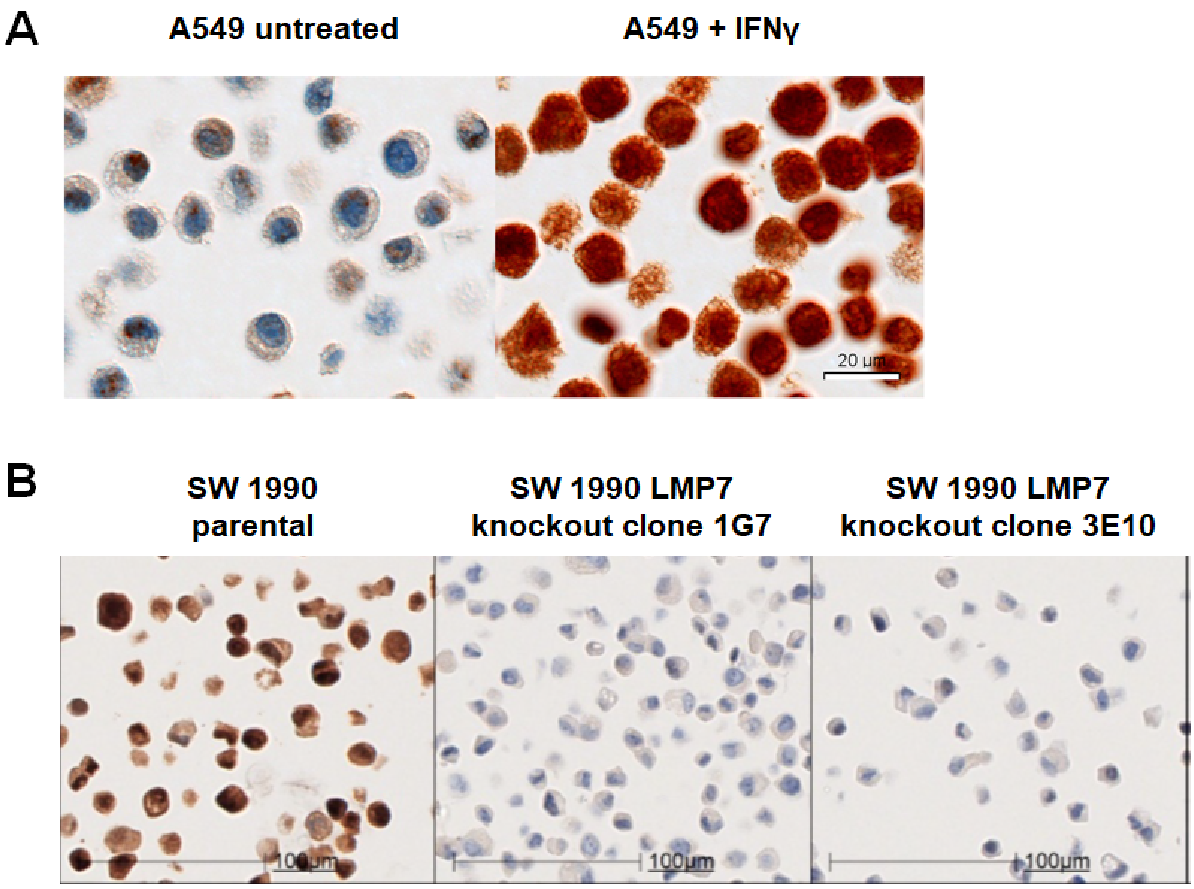

Supplementary Figure S1. Characterization of the specificity of the rabbit anti-human LMP7 monoclonal antibody (clone EPR14482(B)) using human cell lines. A, Immunohistochemistry (IHC) staining using EPR14482(B) in the human lung cancer cell line A549, which was pretreated with or without IFNγ (100 U/mL). **B**, IHC staining using EPR14482(B) in the parental human pancreatic cancer cell line SW 1990, and subclones 1G7 and 3E10 thereof, in which LMP7 (gene name *PSMB8*) had been knocked out using CRISPR targeting.

Supplementary Figure S2

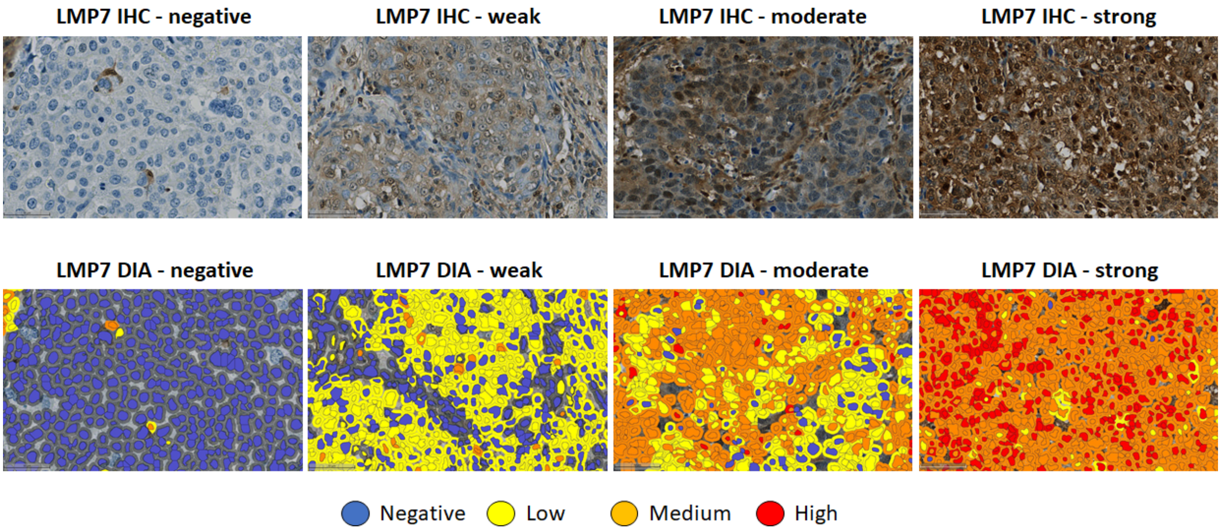

Supplementary Figure S2. Immunohistochemistry (IHC) staining and Digital Image Analysis (DIA) of differential LMP7 expression in TNBC samples. Case QP1359, negative; case QP1368, weak; case QP1363, moderate; case QP1367, strong.

Supplementary Figure S3

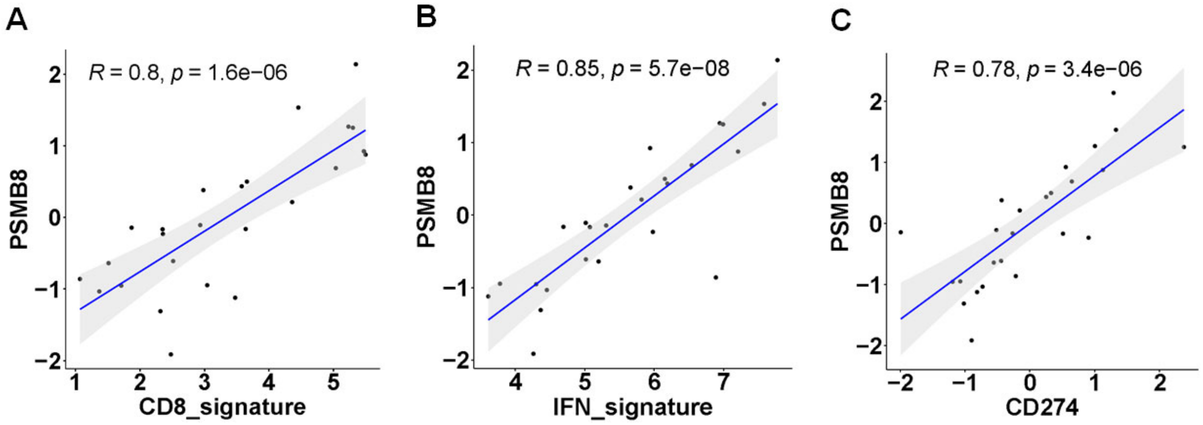

Supplementary Figure S3. Correlation of gene expression of LMP7 with signatures for CD8$^+$ T cells and IFN, and PD-L1 in the TNBC cohort. The TNBC cohort (n = 25) was used to examine correlations of LMP7 (gene name *PSMB8*) with (**A**) CD8$^+$ T cell gene signature, (**B**) IFN gene signature, and (**C**) PD-L1 (gene name *CD274*).

Supplementary Figure S4

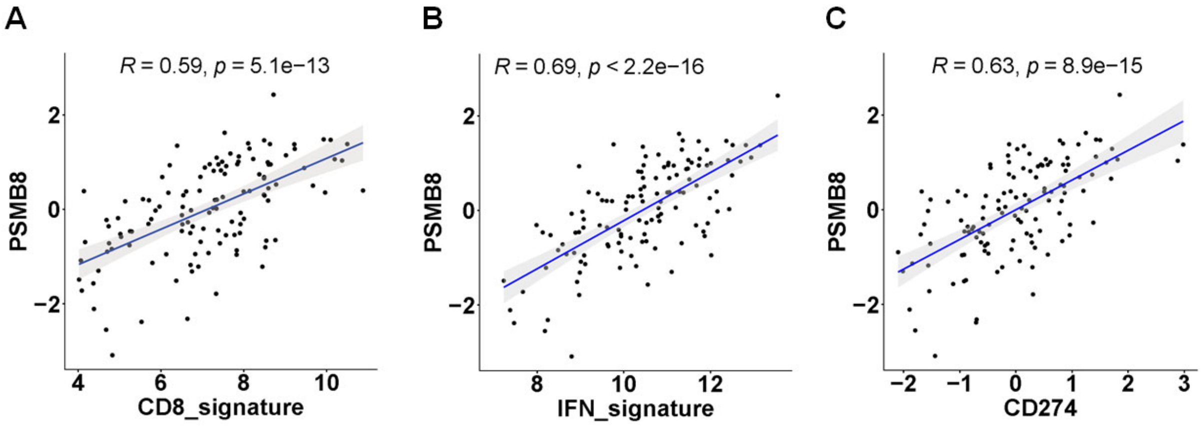

Supplementary Figure S4. Correlation of gene expression of LMP7 with signatures for CD8$^+$ T cells and IFN, and PD-L1 expression in the TCGA TNBC cohort. The TNBC cohort from TCGA (n = 123) was used to examine correlations of LMP7 (gene name *PSMB8*) with (**A**) CD8$^+$ T cell gene signature, (**B**) IFN gene signature, and (**C**) PD-L1 (gene name *CD274*).

Supplementary Figure S5

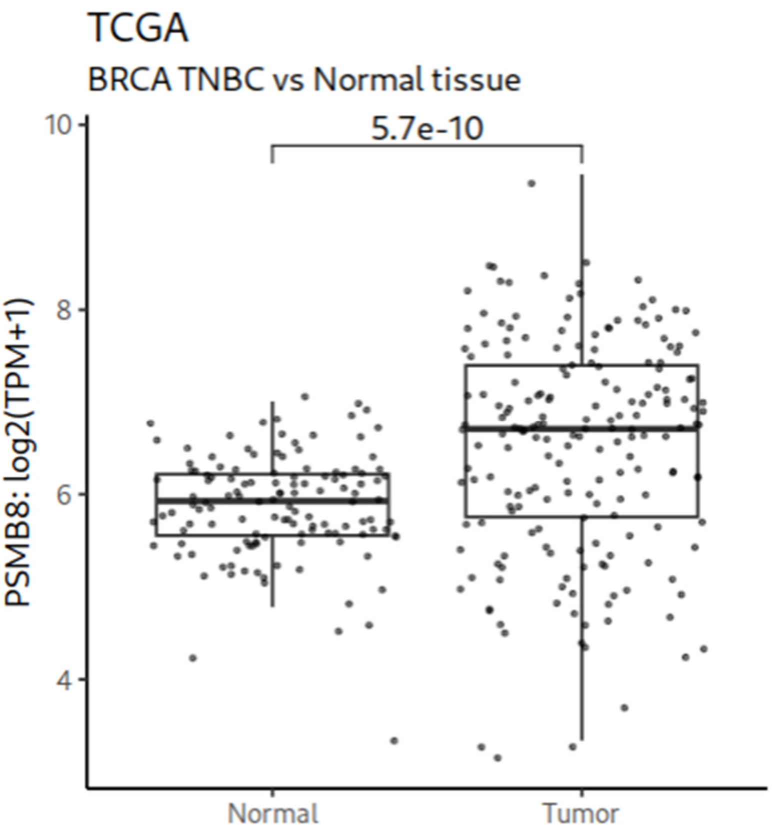

Supplementary Figure S5. Comparison of LMP7 (PSMB8) gene expression between TCGA normal human breast tissue samples and the TCGA TNBC cohort tumor samples.

Supplementary Figure S6

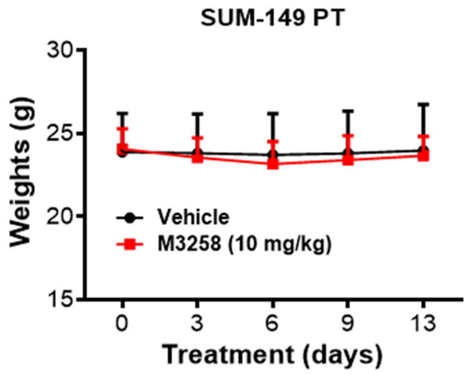

Supplementary Figure S6. Effect of M3258 treatment on mouse body weight. Mean ± standard deviation for mouse body weight data is indicated from the *in vivo* xenograft experiment described in Figure 4A.

Supplementary Figure S7

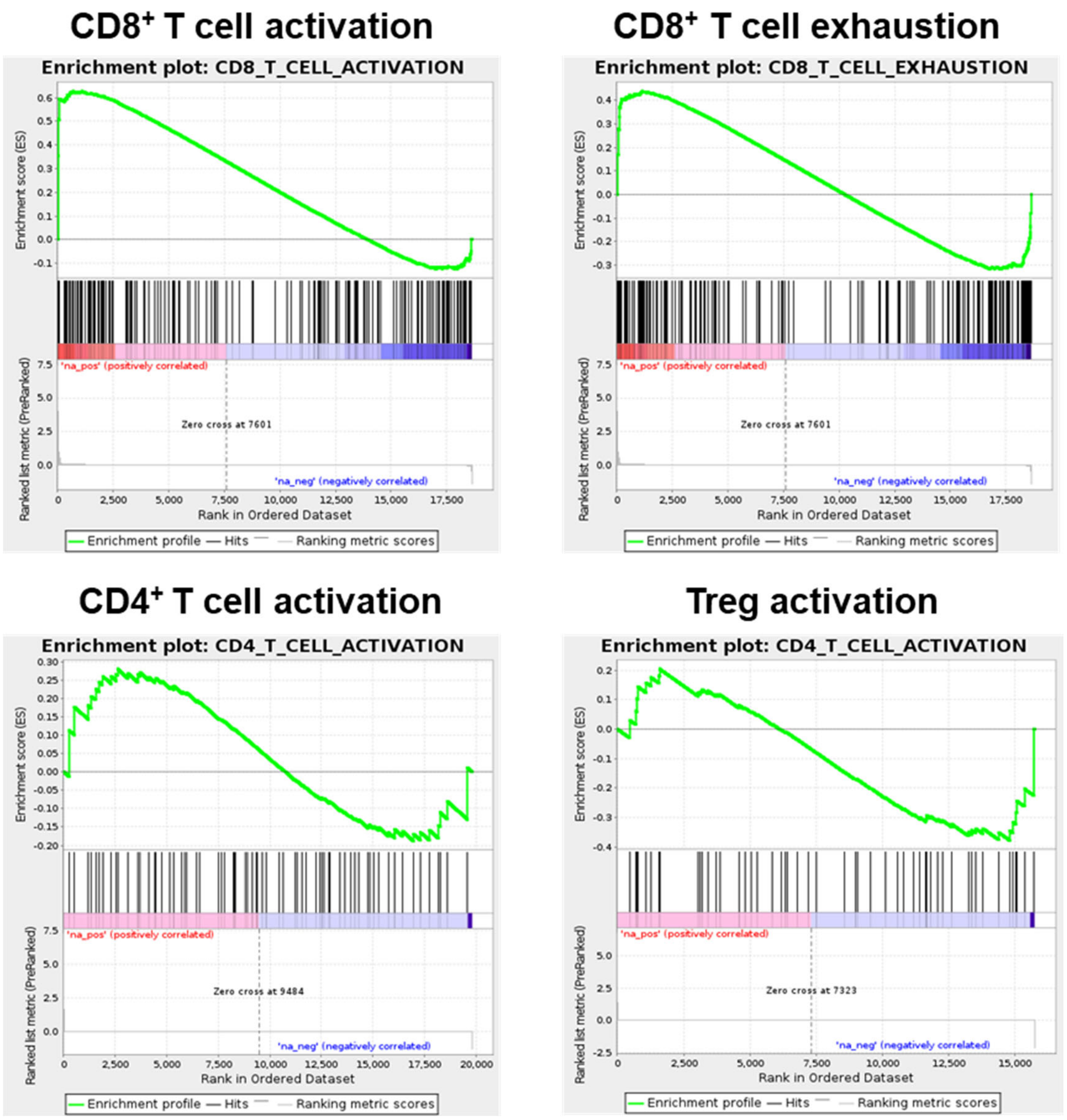

Supplementary Figure S7. Enrichment of pathways regulating activation and exhaustion of CD8^{+} T cells, as well as activation of CD4^{+} T cells and Tregs from SUM-149 PT tumors from mice treated with M3258 versus vehicle control.

Supplementary Tables

Supplementary Table S1. Pathways enriched in the vehicle-treated tumor compartment A versus tumor compartment B (Hallmark).

Pathways	SIZE	ES	NES	NOM p-val	FDR q-val
HALLMARK_EPITHELIAL_MESENCHYMAL_TRANSITION	192	0.73	2.20	0.001	0.001
HALLMARK_E2F_TARGETS	200	0.70	2.14	0.001	0.001
HALLMARK_G2M_CHECKPOINT	197	0.69	2.10	0.001	0.001
HALLMARK_MYC_TARGETS_V1	199	0.67	2.08	0.001	0.001
HALLMARK_MYC_TARGETS_V2	58	0.66	1.72	0.002	0.004
HALLMARK_HEDGEHOG_SIGNALING	34	0.70	1.65	0.007	0.011
HALLMARK_ANGIOGENESIS	35	0.68	1.64	0.013	0.009
HALLMARK_MITOTIC_SPINDLE	198	0.50	1.54	0.001	0.024
HALLMARK_UNFOLDED_PROTEIN_RESPONSE	111	0.54	1.54	0.005	0.024
HALLMARK_MTORC1_SIGNALING	200	0.50	1.52	0.003	0.023
HALLMARK_TNFA_SIGNALING_VIA_NFKB	199	0.49	1.51	0.003	0.026
HALLMARK_HYPOXIA	191	0.49	1.50	0.001	0.026
HALLMARK_INFLAMMATORY_RESPONSE	194	0.49	1.49	0.006	0.028
HALLMARK_KRAS_SIGNALING_UP	191	0.47	1.44	0.003	0.044

Supplementary Table S2. Pathways enriched in the vehicle-treated tumor compartment A versus tumor compartment B (KEGG).

Pathways	SIZE	ES	NES	NOM p-val	FDR q-val
RIBOSOME	131	0.74	2.17	0.001	0.001
CYTOSOLIC_DNA-SENSING_PATHWAY	47	0.71	1.78	0.001	0.023

Supplementary Table S3. Pathways enriched in the vehicle-treated tumor compartment B versus tumor compartment A (Hallmark).

Pathways	SIZE	ES	NES	NOM p-val	FDR q-val
HALLMARK_INTERFERON_ALPHA_RESPONSE	96	-0.55	-1.63	0.002	0.011
HALLMARK_INTERFERON_GAMMA_RESPONSE	196	-0.46	-1.53	0.003	0.017

Supplementary Table S4. Pathways enriched in the vehicle-treated tumor compartment B versus tumor compartment A (KEGG).

Pathways	SIZE	ES	NES	NOM p-val	FDR q-val
ANTIGEN_PROCESSING_AND_PRESENTATION	62	-0.81	-2.31	0.001	0.001
CELL_ADHESION_MOLECULES_(CAMS)	128	-0.66	-2.10	0.001	0.001
PHAGOSOME	140	-0.53	-1.69	0.001	0.019

Supplementary Table S5. Genes affected by M3258 treatment versus vehicle treatment in tumor compartment A.

	log2FoldChange	pvalue	padj	trt.mean	ctr.mean	Regulation
MT1E	-0.72464	8.39E-07	0.0045	2923.348	5126.317	Downregulated
DSG1	1.296237	1.24E-06	0.0045	340.1126	126.227	Upregulated
KRT13	1.734752	2.45E-06	0.004929	56.88476	14.7369	Upregulated
FA2H	1.013734	3.40E-06	0.005218	32.70586	14.40885	Upregulated
AC104532.2	0.560427	4.50E-06	0.005757	131.2775	86.53378	Upregulated
KRT16	0.50813	9.14E-06	0.009884	11582.56	7797.719	Upregulated
CAPNS2	0.712338	1.00E-05	0.010054	617.2046	353.9055	Upregulated
DNM1	0.743732	1.12E-05	0.010458	174.8768	97.84236	Upregulated
TTLL7	0.598343	1.48E-05	0.012232	154.0068	96.21124	Upregulated
RDH12	0.906616	3.24E-05	0.020298	217.5107	105.4074	Upregulated
PIK3R3	-0.75138	3.50E-05	0.020494	639.4129	1166.722	Downregulated
SLC39A2	0.649199	4.22E-05	0.022823	120.5971	72.05749	Upregulated
B4GALNT1	0.611327	4.82E-05	0.025112	87.23532	53.00737	Upregulated
ARRB1	-0.86572	5.26E-05	0.025525	22.2594	45.07358	Downregulated
VTCN1	0.672494	6.19E-05	0.026959	103.1348	60.09852	Upregulated
LRP1B	0.681704	6.26E-05	0.026959	87.46976	51.39687	Upregulated
HOXD10	-1.21758	6.33E-05	0.026959	7.935493	21.25474	Downregulated
ASPG	0.665433	9.75E-05	0.033432	45.95232	26.85785	Upregulated
DYNC1I1	0.775003	0.000111	0.034935	127.6358	67.80268	Upregulated
LNX1	0.618677	0.000118	0.034935	320.7343	192.8036	Upregulated
RNF217-AS1	0.863357	0.000122	0.034935	26.64149	13.65529	Upregulated
DGAT2	0.51213	0.000144	0.038225	760.3748	498.097	Upregulated
GLIS2	-0.53255	0.000177	0.039327	130.1908	204.1271	Downregulated
LINC01752	0.884059	0.000185	0.039327	89.62208	42.82442	Upregulated
VASN	-0.87597	0.000185	0.039327	22.7928	49.28535	Downregulated
SCEL	0.878701	0.000185	0.039327	172.71	83.68864	Upregulated
FAM84A	0.508201	0.000186	0.039327	1228.343	805.7011	Upregulated
IFITM3	-0.59167	0.000237	0.040061	2969.862	4890.175	Downregulated
AIF1L	-0.6934	0.000241	0.040061	29.4711	54.15848	Downregulated
IFITM1	-0.74614	0.000243	0.040061	1196.203	2245.693	Downregulated
HIST1H2BJ	-0.55241	0.000247	0.040061	322.8962	515.6897	Downregulated
NBPF15	0.625663	0.00027	0.040844	40.75107	23.83662	Upregulated

Supplementary Table S6. Genes affected by M3258 treatment versus vehicle treatment in tumor compartment B.

	log2FoldChange	pvalue	padj	trt.mean	ctr.mean	Regulation
SNHG8	-0.65693	4.61E-06	0.005638	64.13203	110.2958	Downregulated
BTG1	-0.77038	1.21E-05	0.005638	115.4277	214.5601	Downregulated
REX1BD	0.621087	1.40E-05	0.005638	108.3267	68.16472	Upregulated
SOX4	-0.76101	2.65E-05	0.006781	187.1073	351.4915	Downregulated
TFCP2L1	0.811097	6.21E-05	0.010894	88.54312	45.33672	Upregulated
NQO1	0.716583	7.89E-05	0.012461	119.4449	65.21936	Upregulated
EREG	-1.25825	0.000154	0.017424	29.98603	87.92681	Downregulated
BLVRB	0.558916	0.000184	0.01769	139.3684	89.32852	Upregulated
NUPR1	-0.60104	0.000202	0.01769	68.59457	112.7983	Downregulated
ACADVL	0.500374	0.000364	0.028742	110.5151	73.7141	Upregulated
PSMC1	0.545391	0.000661	0.0348	71.22685	44.21942	Upregulated
ZFAS1	-0.514	0.000797	0.03814	261.6211	412.8841	Downregulated
TALDO1	0.539014	0.00095	0.03967	195.4577	120.7879	Upregulated

Supplementary Table S7. Major pathways affected by M3258 treatment versus vehicle treatment in tumors (Hallmark).

Effect	Pathways	SIZE	ES	NES	NOM p-val	FDR q-val
Suppressed	HALLMARK_TNFA_SIGNALING_VIA_NFKB	199	-0.73	-1.99	0.001	0.001
	HALLMARK_MYC_TARGETS_V1	199	-0.68	-1.89	0.001	0.004
	HALLMARK_INFLAMMATORY_RESPONSE	196	-0.69	-1.89	0.001	0.003
	HALLMARK_G2Mc_CHECKPOINT	197	-0.65	-1.80	0.001	0.009
	HALLMARK_INTERFERON_GAMMA_RESPONSE	197	-0.65	-1.80	0.001	0.007
	HALLMARK_MYC_TARGETS_V2	58	-0.75	-1.77	0.006	0.008
	HALLMARK_E2F_TARGETS	200	-0.63	-1.73	0.001	0.010
	HALLMARK_WNT_BETA_CATENIN_SIGNALING	40	-0.75	-1.70	0.006	0.015
	HALLMARK_UNFOLDED_PROTEIN_RESPONSE	111	-0.63	-1.63	0.002	0.031
	HALLMARK_IL2_STAT5_SIGNALING	198	-0.56	-1.57	0.008	0.050

Supplementary Table S8. Major pathways affected by M3258 treatment versus vehicle treatment in tumors (KEGG).

Effect	Pathways	SIZE	ES	NES	NOM p-val	FDR q-val
Suppressed	RIBOSOME	132	-0.84	-2.21	0.001	0.001
	ANTIGEN_PROCESSING_AND_PRESENTATION	62	-0.86	-2.03	0.001	0.001
	CELL_ADHESION_MOLECULES_(CAMS)	129	-0.74	-1.94	0.001	0.001
	ERBB_SIGNALING_PATHWAY	83	-0.72	-1.79	0.001	0.028
	JAK-STAT_SIGNALING_PATHWAY	133	-0.66	-1.74	0.002	0.046

Supplementary Table S9. Genes affected by M3258 treatment versus vehicle treatment in CD8$^+$ T cells.

	log2FoldChange	pvalue	padj	trt.mean	ctr.mean	Regulation
UCHL1	7.430227	9.26E-21	8.02E-17	71.3113	0.293337	Upregulated
PSMD11	1.602466	1.40E-15	5.58E-12	100.4062	32.28323	Upregulated
REX1BD	1.332529	1.93E-15	5.58E-12	145.5967	54.59538	Upregulated
MLLT11	2.712766	1.43E-12	3.10E-09	23.63581	4.127958	Upregulated
MAP1A	3.991235	2.87E-11	4.97E-08	12.54582	0.747899	Upregulated
ANXA7	1.106112	2.49E-09	3.09E-06	65.37278	30.47771	Upregulated
PSMB4	1.156172	2.50E-09	3.09E-06	90.6085	41.14324	Upregulated
NAP1L1	0.969717	3.21E-09	3.47E-06	173.0662	87.21527	Upregulated
ACADVL	1.284828	5.69E-09	5.47E-06	70.86262	27.29051	Upregulated
PSMD1	1.307189	6.45E-09	5.58E-06	72.34534	27.70205	Upregulated
UBXN4	0.849912	7.49E-09	5.90E-06	177.3793	93.07677	Upregulated
PSMD12	1.276685	9.02E-09	6.51E-06	54.8453	20.42238	Upregulated
PSMA1	0.898878	1.43E-08	9.51E-06	136.1304	70.39907	Upregulated
B4GALNT1	4.844509	3.59E-08	2.22E-05	8.448154	0.267397	Upregulated
PSMD4	1.012014	4.63E-08	2.67E-05	87.7667	39.50095	Upregulated
SOD1	0.852018	1.43E-07	7.76E-05	216.3699	115.2073	Upregulated
PSMD7	0.965923	2.49E-07	0.000127	111.9311	53.44385	Upregulated
PSMC1	1.128599	3.08E-07	0.000148	79.33	33.7637	Upregulated
PSMD14	1.127016	4.68E-07	0.000213	67.60115	29.15912	Upregulated
TALDO1	0.924102	5.05E-07	0.000219	125.1438	63.65805	Upregulated
NSFL1C	1.227141	7.85E-07	0.000324	34.66201	15.22836	Upregulated
PSMC3	0.926249	8.50E-07	0.000334	77.98995	41.16772	Upregulated
PSMC2	1.164205	1.20E-06	0.000453	54.02306	22.61837	Upregulated
TXNRD1	1.105665	1.41E-06	0.00051	36.74146	14.35494	Upregulated
UFD1	0.859673	3.14E-06	0.001086	73.45199	38.41337	Upregulated
RAD23A	0.849238	3.26E-06	0.001086	73.44551	38.56018	Upregulated
PIR	3.237114	3.69E-06	0.001183	6.843029	0.551614	Upregulated
ADRM1	0.945863	4.49E-06	0.001389	100.1709	48.01499	Upregulated
VCP	0.964451	6.52E-06	0.001947	91.93402	45.61762	Upregulated
PSMB6	0.763195	6.80E-06	0.001962	146.4048	83.92608	Upregulated
FTL	1.132315	7.02E-06	0.001962	975.0736	405.3248	Upregulated
PSMD13	0.720387	1.02E-05	0.002762	78.95483	45.76971	Upregulated
NPLOC4	1.101019	1.25E-05	0.003274	31.3097	13.28022	Upregulated
PSMC6	0.773374	1.36E-05	0.003463	67.34342	37.83466	Upregulated
CCPG1	1.1272	1.41E-05	0.003481	36.36345	15.22216	Upregulated
PSMB2	0.982118	1.49E-05	0.003483	144.0101	66.80448	Upregulated
PSMA3	0.743337	1.49E-05	0.003483	114.4673	63.33274	Upregulated
CYB5R1	1.325381	2.30E-05	0.005235	19.02519	5.962836	Upregulated
AC092683.1	1.272107	3.38E-05	0.007506	26.39254	9.747479	Upregulated
HIST1H4C	-1.42756	3.82E-05	0.008279	42.22277	139.7493	Downregulated
RHBDD3	1.498691	3.98E-05	0.00841	13.42206	3.61503	Upregulated

SELENOK	0.788851	6.54E-05	0.013486	184.4737	93.55173	Upregulated
BRF2	1.670851	7.13E-05	0.014356	11.41702	2.901813	Upregulated
HTATIP2	1.027562	7.88E-05	0.015511	36.80724	15.83424	Upregulated
PSMD6	0.865093	9.13E-05	0.017564	44.96928	22.50065	Upregulated
PRDX1	0.842948	0.000108	0.019822	356.6045	180.8366	Upregulated
PELO	1.065869	0.000126	0.022753	38.98867	15.63956	Upregulated
TXNL1	0.691881	0.00014	0.024799	62.22544	37.99594	Upregulated
PSMD2	0.751061	0.000177	0.03002	83.86461	46.14179	Upregulated
IFITM1	-1.131	0.000179	0.03002	37.40706	97.74625	Downregulated
HINT1	0.682243	0.00018	0.03002	355.3863	205.1536	Upregulated
HMGB2	-0.86075	0.000212	0.034704	77.71082	165.9882	Downregulated
EPSTI1	-1.13618	0.000231	0.036993	18.34969	46.31357	Downregulated
PSMB1	0.555114	0.000244	0.038336	213.1957	136.3071	Upregulated
NEK1	0.992667	0.000256	0.039507	19.97444	8.929641	Upregulated
ZFP36	0.941087	0.000269	0.040838	158.9652	70.35167	Upregulated
ABHD4	2.129197	0.000318	0.04751	5.544809	1.278421	Upregulated
RUFY3	1.108742	0.000337	0.049467	14.17994	5.179553	Upregulated

Supplementary Table S10. Genes affected by M3258 treatment versus vehicle treatment in dendritic cells.

	log2FoldChange	pvalue	padj	trt.mean	ctr.mean	Regulation
ISG20	-1.37133	1.79E-05	0.015899	15.50261	41.49627	Downregulated
GADD45A	-0.61559	2.01E-05	0.015899	125.3624	200.4062	Downregulated
TALDO1	0.71668	3.12E-05	0.018535	95.16627	51.87829	Upregulated
ZFAS1	-0.69834	5.48E-05	0.024455	222.9346	387.4188	Downregulated
TUBA1A	-0.64978	7.40E-05	0.024455	182.6304	308.302	Downregulated
CYTIP	-0.59722	8.07E-05	0.024455	137.3061	219.8737	Downregulated
GPR183	-0.83095	8.23E-05	0.024455	207.8711	403.3885	Downregulated
HLA-DPB1	-0.53649	9.30E-05	0.024576	1418.104	2204.166	Downregulated
IKZF1	-0.72038	0.000127	0.030187	42.86267	78.76868	Downregulated

Supplementary Table S11. Genes affected by M3258 treatment versus vehicle treatment in M1 macrophages.

	log2FoldChange	pvalue	padj	trt.mean	ctr.mean	Regulation
MLLT11	2.906668	3.00E-27	2.20E-23	122.5494	15.39731	Upregulated
TALDO1	1.345764	5.23E-25	1.92E-21	744.2766	287.3604	Upregulated
CCL20	-2.76079	5.42E-17	1.33E-13	9.67473	67.32855	Downregulated
BLVRB	1.352337	2.39E-16	4.38E-13	273.824	101.6724	Upregulated
UCHL1	11.61964	5.10E-15	7.48E-12	129.1636	0	Upregulated
REX1BD	1.050484	8.02E-13	9.81E-10	276.4784	130.4146	Upregulated
CD151	1.404117	3.15E-12	3.30E-09	98.42875	35.28855	Upregulated
AIFM2	2.738234	1.50E-11	1.38E-08	27.68349	3.308964	Upregulated
ACADVL	1.022109	3.67E-11	2.99E-08	292.3127	137.5678	Upregulated
NRN1	1.374637	9.99E-11	7.33E-08	296.8061	111.4273	Upregulated

PSMB2	0.782209	1.41E-10	9.39E-08	300.3745	168.1484	Upregulated
PRDX1	0.724704	2.69E-10	1.65E-07	3760.427	2220.826	Upregulated
PSMD11	1.099514	7.14E-10	4.03E-07	167.6741	73.57222	Upregulated
AIF1	-0.88451	8.11E-10	4.25E-07	840.151	1611.078	Downregulated
PSMB7	0.785053	9.01E-10	4.41E-07	248.0743	136.6728	Upregulated
PIR	2.746965	1.06E-09	4.88E-07	23.49702	2.622999	Upregulated
VCP	0.688664	9.83E-09	4.24E-06	201.4019	120.9495	Upregulated
TXN	1.215243	1.09E-08	4.46E-06	1177.914	478.2837	Upregulated
APOL2	1.660845	2.26E-08	8.51E-06	46.28159	13.51376	Upregulated
LDLRAD4	-1.01144	2.32E-08	8.51E-06	95.71281	200.2253	Downregulated
CCDC189	1.891394	3.17E-08	1.11E-05	50.38075	13.44875	Upregulated
SOD1	0.797875	4.00E-08	1.34E-05	398.06	216.4978	Upregulated
PRSS56	1.384744	5.42E-08	1.65E-05	58.27734	21.37823	Upregulated
RPS4X	-0.63609	5.74E-08	1.65E-05	1771.761	2844.454	Downregulated
PSMB4	0.753468	5.85E-08	1.65E-05	229.2166	131.4042	Upregulated
PSMD2	0.753566	6.60E-08	1.79E-05	193.9388	110.3378	Upregulated
NFKBIA	-0.92045	1.10E-07	2.89E-05	484.1603	965.9424	Downregulated
PSMC4	0.929866	1.52E-07	3.72E-05	234.0331	120.3515	Upregulated
SPART	1.739152	1.80E-07	4.27E-05	28.58511	7.551511	Upregulated
PSMA3	0.649362	2.08E-07	4.77E-05	248.4448	151.5361	Upregulated
SMYD3	0.9627	2.74E-07	6.09E-05	210.6336	102.8476	Upregulated
PSMB6	0.670709	3.92E-07	8.46E-05	432.0525	261.4699	Upregulated
C1orf54	1.202521	7.66E-07	0.000161	117.1122	46.74495	Upregulated
EGOT	-1.51947	1.02E-06	0.000208	14.29315	45.38646	Downregulated
LIMCH1	2.050484	1.27E-06	0.000253	19.13432	4.110693	Upregulated
RGS16	-1.39089	1.31E-06	0.000253	116.6287	339.3558	Downregulated
PSMD14	0.709497	1.35E-06	0.000254	146.3355	85.53625	Upregulated
CD207	0.985135	1.86E-06	0.000341	1535.371	720.2947	Upregulated
CSTA	-1.60523	2.32E-06	0.000415	15.69346	58.34991	Downregulated
PSMC3	0.599347	2.40E-06	0.000419	192.2288	122.1252	Upregulated
PSMA5	0.593929	2.83E-06	0.000476	234.1773	146.3013	Upregulated
UFD1	0.690811	2.86E-06	0.000476	140.2664	80.13991	Upregulated
PHETA1	0.911629	3.58E-06	0.000584	86.26642	44.84136	Upregulated
FERMT3	0.64968	4.62E-06	0.000736	129.1919	77.37144	Upregulated
CYB5R1	1.285643	4.91E-06	0.000766	149.7461	55.86965	Upregulated
IL1RN	-0.7387	6.16E-06	0.000886	351.6345	618.0893	Downregulated
GTF2B	-0.68857	6.45E-06	0.000911	72.22334	121.1768	Downregulated
PSMD1	0.842794	7.47E-06	0.001034	142.7597	72.88154	Upregulated
SLC29A1	0.999975	8.03E-06	0.001091	180.6698	83.30184	Upregulated
PSMC6	0.720537	8.57E-06	0.001143	147.3036	84.41352	Upregulated
PSMC1	0.555819	1.04E-05	0.001344	195.0671	125.5131	Upregulated
S100A2	-1.35488	1.11E-05	0.001408	55.76934	160.7554	Downregulated
PSAP	0.809529	1.22E-05	0.00152	249.2809	132.1638	Upregulated

ANXA7	0.626417	1.30E-05	0.001574	151.7805	94.49909	Upregulated
SH3BP5	1.144456	1.31E-05	0.001574	81.31071	31.83102	Upregulated
MAP1A	5.385742	1.35E-05	0.001595	23.02799	0.20858	Upregulated
PELO	1.820941	1.45E-05	0.001689	21.65782	4.947466	Upregulated
UBB	-0.71566	2.10E-05	0.002405	400.5285	706.8423	Downregulated
HINT1	0.565551	2.71E-05	0.003062	1247.486	800.5849	Upregulated
GRASP	-1.07283	2.76E-05	0.003066	73.21144	167.6992	Downregulated
TCN2	1.498737	3.03E-05	0.003207	30.99152	10.82911	Upregulated
CCPG1	0.973879	3.03E-05	0.003207	82.39866	38.39134	Upregulated
PSMA1	0.510797	3.08E-05	0.003207	283.327	191.3802	Upregulated
ACOT13	0.732077	3.08E-05	0.003207	133.0529	75.34476	Upregulated
RPS27	-0.53382	3.12E-05	0.003207	1447.898	2197.976	Downregulated
APOL1	1.724174	3.15E-05	0.003207	25.63997	6.44692	Upregulated
FTL	0.831304	3.45E-05	0.003423	9774.739	5039.325	Upregulated
RPS6	-0.56952	3.51E-05	0.003431	2195.056	3444.221	Downregulated
TCEAL3	-1.12816	3.62E-05	0.003497	14.03711	34.17353	Downregulated
PSMA2	0.755518	3.72E-05	0.003544	69.51705	38.21952	Upregulated
BCAP31	0.655446	4.09E-05	0.003845	192.1608	113.0521	Upregulated
RAB5IF	0.74293	4.85E-05	0.004392	93.67591	51.73636	Upregulated
HLA-DOA	-0.8856	5.21E-05	0.004586	24.67101	49.80398	Downregulated
EMP3	0.567246	5.25E-05	0.004586	681.8109	434.076	Upregulated
SLC16A7	1.406268	5.64E-05	0.004735	20.35275	5.928687	Upregulated
SFN	-1.16683	5.66E-05	0.004735	34.61466	88.23062	Downregulated
NR4A3	-0.76853	5.66E-05	0.004735	153.4115	277.7308	Downregulated
PSENEN	0.732427	5.72E-05	0.004735	87.20611	47.63258	Upregulated
UBXN4	0.574138	5.74E-05	0.004735	295.18	184.1336	Upregulated
RTN3	0.683082	5.84E-05	0.004765	204.2893	117.7881	Upregulated
PSMD6	0.74818	5.99E-05	0.004833	95.07196	51.74677	Upregulated
AKR1B1	-0.96011	6.07E-05	0.004839	22.85564	48.71901	Downregulated
NAP1L1	0.538193	6.89E-05	0.00542	343.4469	224.0662	Upregulated
FKBP11	0.850124	6.94E-05	0.00542	47.82123	22.7293	Upregulated
ADRM1	0.624541	7.31E-05	0.005645	152.607	90.15212	Upregulated
PABPC1	-0.50996	7.69E-05	0.005875	315.3103	473.8167	Downregulated
MZT2B	-0.66149	9.37E-05	0.006946	140.7275	241.4459	Downregulated
JOSD2	0.608418	0.000101	0.007396	88.69818	54.09586	Upregulated
FBL	-0.75393	0.000104	0.007452	37.32554	70.33499	Downregulated
NINJ1	-0.90042	0.000105	0.007452	23.25096	45.44915	Downregulated
SEMA7A	-1.32945	0.00011	0.007741	15.70368	44.35469	Downregulated
CD5L	1.400924	0.00012	0.008385	38.18042	11.35295	Upregulated
TNFAIP2	1.310876	0.000123	0.008416	33.37562	12.03034	Upregulated
TSPO	0.549544	0.000123	0.008416	1044.478	668.1304	Upregulated
GPR65	-0.80153	0.000127	0.008646	48.28139	92.29048	Downregulated
ANKRD37	-0.97507	0.00013	0.008667	26.09252	56.09121	Downregulated

SAMD9L	1.00222	0.000137	0.008958	169.8125	73.3109	Upregulated
ASAH1	0.615857	0.000138	0.008987	81.18063	49.51795	Upregulated
NRIP3	1.60906	0.00014	0.009028	17.71229	4.724081	Upregulated
LSP1	-0.9246	0.000148	0.009428	31.11189	66.24576	Downregulated
KRT17	-0.9655	0.000168	0.010621	219.5288	487.3518	Downregulated
HTATIP2	0.866302	0.000181	0.011207	71.26843	34.63495	Upregulated
GABARAPL1	1.172759	0.000182	0.011207	32.84391	12.66296	Upregulated
DUSP2	-0.69547	0.000187	0.011456	235.3655	412.9238	Downregulated
HES1	-1.0915	0.0002	0.012129	27.29011	72.78248	Downregulated
SNCA	1.027126	0.000203	0.012161	63.05255	27.34097	Upregulated
PSMB5	0.585017	0.000204	0.012161	149.9708	95.59554	Upregulated
FLOT1	-0.5899	0.000216	0.012732	85.51405	134.9967	Downregulated
IFITM2	-1.49483	0.000249	0.01415	4.472509	16.00164	Downregulated
TFEC	1.050554	0.000266	0.015016	42.90373	18.29359	Upregulated
RPL23A	-0.56341	0.000271	0.015073	876.9593	1397.456	Downregulated
MAP1LC3A	-0.72827	0.000272	0.015073	27.59456	51.61814	Downregulated
CAPN2	0.71377	0.000273	0.015073	74.18745	40.17739	Upregulated
AAMDC	0.865378	0.00029	0.015854	56.99341	26.50459	Upregulated
PSMD12	0.676013	0.000341	0.017964	72.06551	42.08309	Upregulated
CDK6	0.715844	0.000343	0.017964	50.08476	26.39812	Upregulated
MALAT1	0.604282	0.000349	0.018175	46411.89	27998.3	Upregulated
RPL36	-0.53689	0.000359	0.018509	758.2054	1189.632	Downregulated
CCR6	0.629031	0.000372	0.018935	158.2028	93.98709	Upregulated
GPR183	-0.79512	0.000374	0.018941	530.4582	1041.457	Downregulated
PSMC2	0.514249	0.000389	0.019432	105.782	70.26231	Upregulated
SELENOM	-0.84579	0.000403	0.019847	18.66727	36.70584	Downregulated
TRAF1	-0.75056	0.000406	0.019847	38.57264	70.29134	Downregulated
TNFSF13B	1.872377	0.000411	0.019946	21.94123	4.055498	Upregulated
SH2D3A	-1.35083	0.000434	0.020807	6.172622	17.23132	Downregulated
SHKBP1	0.578187	0.000461	0.021913	67.33458	39.80616	Upregulated
THUMPD3	0.692609	0.000466	0.021913	42.28257	22.83894	Upregulated
CD99	0.511846	0.000476	0.022244	282.3291	183.2514	Upregulated
CD163L1	1.072558	0.000479	0.022244	20.85735	8.304251	Upregulated
RFLNB	-1.0562	0.000512	0.023638	12.7814	34.47128	Downregulated
HSD17B4	0.579238	0.000516	0.023652	67.67641	42.77882	Upregulated
PILRA	1.608061	0.00052	0.023677	18.78317	4.392949	Upregulated
BCL11A	-1.11128	0.000538	0.024239	13.63195	33.82132	Downregulated
TBC1D4	-0.57588	0.000538	0.024239	229.8497	371.1687	Downregulated
NOL3	0.815034	0.000554	0.024384	31.15489	15.77669	Upregulated
TNFSF9	-0.87196	0.000576	0.024987	14.14114	30.07369	Downregulated
MZT2A	-0.60632	0.000585	0.025257	93.07688	156.1402	Downregulated
RPS27L	0.534042	0.00059	0.025322	222.1935	140.4009	Upregulated
HILPDA	-0.96066	0.000648	0.027488	14.80384	34.97084	Downregulated

ABHD3	0.923706	0.000662	0.027642	35.68993	14.34384	Upregulated
MB21D2	-0.77712	0.000663	0.027642	21.99518	42.06966	Downregulated
MPHOSPH10	0.545694	0.000678	0.02809	62.0087	38.96892	Upregulated
RAB13	-0.86146	0.000713	0.029372	18.20074	37.16865	Downregulated
SLC39A13	0.659072	0.000738	0.030245	54.67396	29.97593	Upregulated
MACC1	0.759539	0.000762	0.03105	37.97584	20.17948	Upregulated
CD37	-0.50732	0.000771	0.031241	76.21658	113.618	Downregulated
TNFAIP6	-0.98387	0.000816	0.032907	23.367	53.33282	Downregulated
ALCAM	0.642116	0.000836	0.033537	216.608	123.0944	Upregulated
CTSD	0.608283	0.000886	0.034879	326.0649	189.1284	Upregulated
PPIF	-0.56863	0.000907	0.035369	114.6076	187.4602	Downregulated
HLA-DQA2	-0.67738	0.000924	0.035674	972.1752	1777.478	Downregulated
FOSL2	-0.68495	0.000952	0.036479	42.85842	74.33278	Downregulated
SERPINB9	-0.76265	0.000955	0.036479	53.15569	104.9003	Downregulated
STK38L	-0.58723	0.000976	0.037105	38.8825	61.23978	Downregulated
CD55	-0.51976	0.000987	0.037329	66.02971	104.3018	Downregulated
RHBDD3	1.05042	0.001071	0.039692	18.85446	6.557441	Upregulated
SPTAN1	0.7046	0.001086	0.039731	43.76871	21.75673	Upregulated
OPN3	-0.57761	0.001088	0.039731	40.65746	64.8498	Downregulated
HMGN5	0.594674	0.001148	0.041496	55.66766	35.16651	Upregulated
ZFAS1	-0.58589	0.001199	0.042896	160.5684	271.0071	Downregulated
RCN3	-1.05504	0.001222	0.043296	8.850431	21.41186	Downregulated
RDH11	0.620247	0.001312	0.046297	62.17194	35.47982	Upregulated
ARL5B	-0.96483	0.001401	0.048714	14.08154	32.13009	Downregulated
PER3	0.730104	0.001432	0.049337	31.05578	16.79124	Upregulated
PAFAH1B3	-0.83533	0.001457	0.049952	22.96269	50.4153	Downregulated

Supplementary Table S12. Genes affected by M3258 treatment versus vehicle treatment in M2 macrophages.

	log2FoldChange	pvalue	padj	trt.mean	ctr.mean	Regulation
UCHL1	8.433964	1.95E-18	3.57E-15	49.44693	0.093455	Upregulated
TALDO1	1.167485	1.57E-06	0.001436	143.8971	63.64364	Upregulated
BLVRB	1.377772	7.72E-06	0.004718	49.48113	21.12019	Upregulated
CTSK	3.14261	1.61E-05	0.006538	32.77131	2.371321	Upregulated
PLD3	1.46401	1.99E-05	0.006538	116.538	41.18873	Upregulated
UBE2S	-1.43583	2.14E-05	0.006538	10.62559	36.95387	Downregulated
SPTAN1	1.676151	3.54E-05	0.009267	30.31183	8.484004	Upregulated
IL1B	-1.86043	4.19E-05	0.009617	17.33691	106.8011	Downregulated
HIST1H4C	-2.38971	5.11E-05	0.010423	4.068153	44.88865	Downregulated
ATF3	1.484441	6.04E-05	0.011078	117.4647	36.49153	Upregulated
NCF1	2.598339	8.08E-05	0.01287	31.97755	4.609871	Upregulated
REX1BD	1.021547	8.42E-05	0.01287	57.05843	30.3647	Upregulated
C1orf54	1.66253	9.15E-05	0.012913	36.4636	11.79839	Upregulated
FTL	1.065154	0.000131	0.0161	8599.885	3612.524	Upregulated

CLEC7A	0.922581	0.000132	0.0161	120.8487	59.84032	Upregulated
MKI67	-1.85644	0.000173	0.019774	6.462975	30.23706	Downregulated
CXCL2	1.252453	0.000236	0.025511	208.4432	77.29551	Upregulated
ACP5	1.183498	0.000271	0.027616	79.03799	36.04277	Upregulated
GPX3	1.302951	0.000309	0.029024	43.44666	18.29994	Upregulated
RPS2	-0.71359	0.000331	0.029024	282.4107	528.0604	Downregulated
ABCC3	1.175768	0.000332	0.029024	21.27093	9.371727	Upregulated
PSMB6	0.784822	0.000373	0.030538	91.93376	52.3312	Upregulated
LGMN	1.867785	0.000408	0.030538	245.7159	49.92862	Upregulated
GM2A	1.092373	0.000413	0.030538	31.79084	14.42485	Upregulated
STAT1	1.80664	0.000459	0.032409	92.89048	19.98956	Upregulated
IL18	1.09703	0.000501	0.03263	41.21938	16.63611	Upregulated
APLP2	0.826109	0.000506	0.03263	88.01525	50.48664	Upregulated
CTSZ	1.362952	0.000516	0.03263	824.9359	261.7016	Upregulated
ECM1	1.15823	0.000588	0.035951	29.96554	11.85158	Upregulated
SQSTM1	0.965953	0.000629	0.037184	95.20118	42.92016	Upregulated
RPL35	-0.74663	0.000659	0.037759	92.96348	204.0721	Downregulated
IL6	1.615844	0.000689	0.037825	37.08826	7.93515	Upregulated
ASAH1	0.953738	0.000701	0.037825	64.50228	31.34365	Upregulated
GLA	1.058031	0.000725	0.038015	56.03984	25.22743	Upregulated
GLRX	0.913038	0.00077	0.03844	90.28662	34.6974	Upregulated
LAMP2	0.940879	0.000776	0.03844	41.92958	19.43492	Upregulated
BNIP3	1.411038	0.000896	0.043238	26.4265	9.076705	Upregulated
ATP6V1A	0.974843	0.000972	0.044569	28.73769	11.72587	Upregulated
CCPG1	1.303258	0.001029	0.046039	23.66098	9.721346	Upregulated

Supplementary Table S13. Major pathways affected by M3258 treatment versus vehicle treatment in immune cells (Hallmark).

Immune Cells	Effect	Pathways	SIZE	ES	NES	NOM p-val	FDR q-val
**CD8⁺ T cells**	Suppressed	HALLMARK_INTERFERON_ALPHA_RESPONSE	95	-0.75	-2.04	0.001	0.006
**M1 macrophages**	Suppressed	HALLMARK_TNFA_SIGNALING_VIA_NFKB	196	-0.71	-1.87	0.001	0.009
**M2 macrophages**	Suppressed	HALLMARK_E2F_TARGETS	200	-0.81	-2.24	0.001	0.001
		HALLMARK_G2M_CHECKPOINT	196	-0.77	-2.16	0.001	0.001
		HALLMARK_MYC_TARGETS_V1	199	-0.61	-1.71	0.001	0.025
		HALLMARK_MYC_TARGETS_V2	58	-0.62	-1.57	0.089	0.040

Supplementary Table S14. Major pathways affected by M3258 treatment versus vehicle treatment in immune cells (KEGG).

Immune Cells	Effect	Pathways	SIZE	ES	NES	NOM p-val	FDR q-val
**CD8⁺ T cells**	Suppressed	RIBOSOME	131	-0.77	-2.18	0.001	0.003
**M1 macrophages**	Suppressed	RIBOSOME	130	-0.88	-2.23	0.001	0.001
	Enriched	PROTEASOME	40	0.96	1.96	0.001	0.011

**M2 macrophages**	Suppressed	RIBOSOME	129	-0.93	-2.52	0.001	0.001
**Dendritic cells**	Suppressed	RIBOSOME	128	-0.91	-2.05	0.001	0.003

Supplementary Table S15. Genes affected by M3258 treatment versus vehicle treatment in CD8$^+$ T cells.

Gene	log2FoldChange	pvalue	padj	trt.mean	ctr.mean	Regulation
**UCHL1**	7.430227	9.26E-21	8.02E-17	71.3113	0.293337	Upregulated
